# Association of Socioeconomic Status and Infarct Volume With Functional Outcome in Patients With Ischemic Stroke

**DOI:** 10.1001/jamanetworkopen.2022.9178

**Published:** 2022-04-27

**Authors:** Ahmed Ghoneem, Michael T. Osborne, Shady Abohashem, Nicki Naddaf, Tomas Patrich, Tawseef Dar, Amr Abdelbaky, Adeeb Al-Quthami, Jason H. Wasfy, Katrina A. Armstrong, Hakan Ay, Ahmed Tawakol

**Affiliations:** 1 Cardiovascular Imaging Research Center, Massachusetts General Hospital and Harvard Medical School, Boston; 2 Cardiology Division, Massachusetts General Hospital and Harvard Medical School, Boston; 3 Department of Medicine, Massachusetts General Hospital and Harvard Medical School, Boston; 4 Anithoula A. Martinos Center for Biomedical Imaging, Massachusetts General Hospital and Harvard Medical School, Boston; 5 Takeda Pharmaceutical Company Limited, Cambridge, Massachusetts

## Abstract

**Importance:**

Long-term disability after stroke is associated with socioeconomic status (SES). However, the reasons for such disparities in outcomes remain unclear.

**Objective:**

To assess whether lower SES is associated with larger admission infarct volume and whether initial infarct volume accounts for the association between SES and long-term disability.

**Design, Setting, and Participants:**

This cohort study was conducted in a prospective, consecutive population (n = 1256) presenting with acute ischemic stroke who underwent magnetic resonance imaging (MRI) within 24 hours of admission. Patients were recruited in Massachusetts General Hospital, Boston, from May 31, 2009, to December 31, 2011. Data were analyzed from May 1, 2019, until June 30, 2020.

**Main Outcomes and Measures:**

Initial stroke severity (within 24 hours of presentation) was determined using clinical (National Institutes of Health Stroke Scale [NIHSS]) and imaging (infarct volume by diffusion-weighted MRI) measures. Stroke etiologic subtypes were determined using the Causative Classification of Ischemic Stroke algorithm. Long-term stroke disability was measured using the modified Rankin Scale. Socioeconomic status was estimated using zip code–derived median household income and census block group–derived area deprivation index (ADI). Regression and mediation analyses were performed.

**Results:**

A total of 1098 patients had imaging and SES data available (mean [SD] age, 68.1 [15.7] years; 607 men [55.3%]). Income was inversely associated with initial infarct volume (standardized β, −0.074 [95% CI, −0.127 to −0.020]; *P* = .007), initial NIHSS (standardized β, −0.113 [95% CI, −0.171 to −0.054]; *P* < .001), and long-term disability (standardized β, −0.092 [95% CI, −0.149 to −0.035]; *P* = .001), which remained significant after multivariable adjustments. Initial stroke severity accounted for 64% of the association between SES and long-term disability (standardized β, −0.063 [95% CI, −0.095 to −0.029]; *P* < .05). Findings were similar when SES was alternatively assessed using ADI.

**Conclusions and Relevance:**

The findings of this cohort study suggest that lower SES is associated with larger infarct volumes on presentation. These SES-associated differences in initial stroke severity accounted for most of the subsequent disparities in long-term disability in this study. These findings shift the culpability for SES-associated disparities in poststroke disability from poststroke factors to those that precede presentation.

## Key Points

**Question:**

Is socioeconomic status (SES) independently associated with infarct size, clinical severity, and long-term functional outcomes in patients with ischemic stroke?

**Findings:**

In this single-center cohort study of 1098 consecutive patients with ischemic stroke, initial stroke severity was assessed using magnetic resonance imaging of the brain and clinical indexes. Socioeconomic status (measured as median neighborhood income and area deprivation index) was independently associated with imaging and clinical measures of initial stroke severity, which in turn accounted for 64% of the association between SES and long-term functional outcome.

**Meaning:**

These findings suggest that individuals with lower SES have worse long-term functional outcomes, primarily because lower SES is associated with larger infarcts and more clinically severe strokes on presentation, even after adjusting for differences in risk factors and therapies.

## Teaser

This single-center cohort study assesses the association between socioeconomic status (SES) and infarct volume in patients presenting with with acute ischemic stroke and investigates the potential mediating role of infarct volume in the association between SES and long-term disbility.

## Introduction

Stroke is a common cause of death^1^ and disability.^2^ Low socioeconomic status (SES) has repeatedly been shown to be associated with worse stroke outcomes, including poorer functional outcomes and short-term mortality.^3,4^ Such SES-related disparities in outcome may result from differences in comorbidities, risk factors, or access to care.^5^ However, those factors alone do not sufficiently explain the observed variance. A better understanding of mechanisms linking SES to disparities in stroke outcomes is needed.

An alternative hypothesis is that lower SES is associated with larger infarct volumes on presentation (ie, before care delivery) and that such differences in initial stroke size may largely account for disparities in outcomes. However, the few prior studies that investigated this hypothesis^6,7,8,9,10,11^ relied on clinical scales of stroke severity (ie, National Institutes of Health Stroke Scale [NIHSS]) and produced inconsistent findings. Although the NIHSS estimates both short- and long-term outcomes,^12^ it is not without limitations in evaluating initial stroke severity.^13,14,15,16^ Accordingly, prior studies that rely exclusively on the NIHSS may lack adequate precision to fully assess associations between SES and outcome. Alternatively, initial stroke severity can be objectively quantified as infarct volume using magnetic resonance imaging (MRI) to yield a reproducible and prognostic measure of biological injury.^17,18,19^ Several groups have found that adding infarct volume to NIHSS improves outcome estimates, suggesting that imaging and clinical metrics provide independent information associated with outcomes.^17,20,21,22^ Thus, infarct volume represents an important tissue-level marker that complements clinical scores for assessment of stroke severity and is well suited for proof-of-concept studies.

To investigate the association between SES and stroke outcomes, we studied a cohort of consecutive patients with ischemic stroke and assessed their initial stroke severity (using clinical and MRI-based measures) and long-term disability. We tested the hypotheses that (1) lower SES is independently associated with higher initial stroke severity (measured as admission NIHSS score and infarct volume) and (2) the association between SES and long-term functional outcomes is mediated by initial stroke severity.

## Methods

### Data Collection

Data were prospectively collected from consecutive patients admitted to Massachusetts General Hospital, Boston, with acute ischemic stroke within 72 hours of symptom onset based on clinical assessment and computed tomography findings. The parent study, the National Institutes of Health–funded Heart-Brain Interactions Study, was conducted in a population recruited from May 31, 2009, to December 31, 2011 (eFigure 1 in the Supplement). The parent study’s exclusion criteria were contraindications to MRI or lack of evaluable MRI images. For this SES substudy, patients were excluded if their SES data could not be derived (ie, no US-based home address). The Mass General Brigham Institutional Review Board approved the study. Each patient provided written or oral informed consent. Data were analyzed from May 1, 2019, to June 30, 2020. This study followed the Strengthening the Reporting of Observational Studies in Epidemiology (STROBE) reporting guideline.

### Assessment of Home Addresses and SES

Individual addresses were collected on admission from the medical records. The primary SES measure was local median household income using zip codes, which were estimated from the US Census Bureau Fact Finder’s 2016 American Community Survey.^23^ The secondary SES measure was the area deprivation index (ADI), calculated at the census block group level, the closest approximation to a neighborhood (eMethods 1 in the Supplement).^24^

### Assessment of Infarct Volume by MRI

An MRI was obtained from each patient for clinical evaluation. Diffusion-weighted imaging (b values, 0 and 1000 s/mm^2^) was performed on two 1.5T instruments (General Electric Company and Siemens AG). Expert manual annotations of acute infarcts on diffusion-weighted imaging were generated by investigators blinded to SES data using image outlining software (MRIcron [NITRC]). All outlines were adjudicated by a senior stroke neurologist (H.A.) (eMethods 2 in the Supplement).

### Determination of Etiologic Mechanism of Stroke

Stroke etiology was determined using the Causative Classification of Ischemic Stroke,^25,26^ a semiautomated, evidence-based algorithm that incorporates information from each patient’s diagnostic evaluation to categorize stroke etiology into 1 of 5 major subtypes (eMethods 3 in the Supplement).^27^ These subtypes are (1) supra-aortic large-artery atherosclerosis (LAA), (2) cardioembolism (CE), (3) small-artery occlusion, (4) other uncommon causes (eg, acute arterial dissection, cerebral vasculitis, cerebral venous thrombosis, and acute disseminated intravascular coagulation), and (5) undetermined causes. The algorithm maximizes interexaminer reliability in stroke classification.^28^

### Clinical Data

Clinical data were collected at presentation, including demographic characteristics, admission stroke severity (ie, NIHSS score),^12^ medications, and cerebrovascular risk factors. Race and ethnicity data were derived from the medical record as provided by each patient. Postdischarge outcome data were collected by medical record review using a standard data collection manual. Assessment of long-term disability was performed at a mean (SD) of 90 (15) days using the modified Rankin Scale score.^29^ A blinded investigator performed outcome assessments through in-person evaluations, telephone interviews, or outpatient physician note review.

### Statistical Analysis

Statistical analysis was performed using SPSS, version 25 (IBM Corporation). Normality was assessed by the Shapiro-Wilk test. Bivariate correlations were assessed using the Pearson and Spearman methods as appropriate. We used χ^2^ tests and univariable linear regression for comparisons across SES quintiles for categorical and continuous variables, respectively. Linear regression was used to evaluate the association between SES and clinical and imaging measures of stroke severity indexes as well as 90-day disability (eMethods 4 in the Supplement). Patients with missing data were excluded from corresponding analyses. Mediation analysis was performed to test whether SES exerts its association with poststroke disability via the hypothesized mediators (ie, infarct volume and NIHSS score), either singularly or in a series (eMethods 5 in the Supplement). Median income, infarct volume, NIHSS score, modified Rankin Scale score, and symptom duration were used as standardized continuous variables. Important covariables were determined a priori and included demographic factors (ie, age, race and ethnicity, sex), stroke risk factors (ie, hypertension, diabetes, hyperlipidemia, atrial fibrillation, current smoking, coronary artery disease, congestive heart failure, and prior stroke or transient ischemic attack),^30^ prestroke medications (eg, antiplatelet drugs, anticoagulants, and statins), discharge medications, stroke symptom duration, and insurance status. Statistical significance was determined as 2-sided *P* < .05.

## Results

### Patient Characteristics

**Figure 1.**
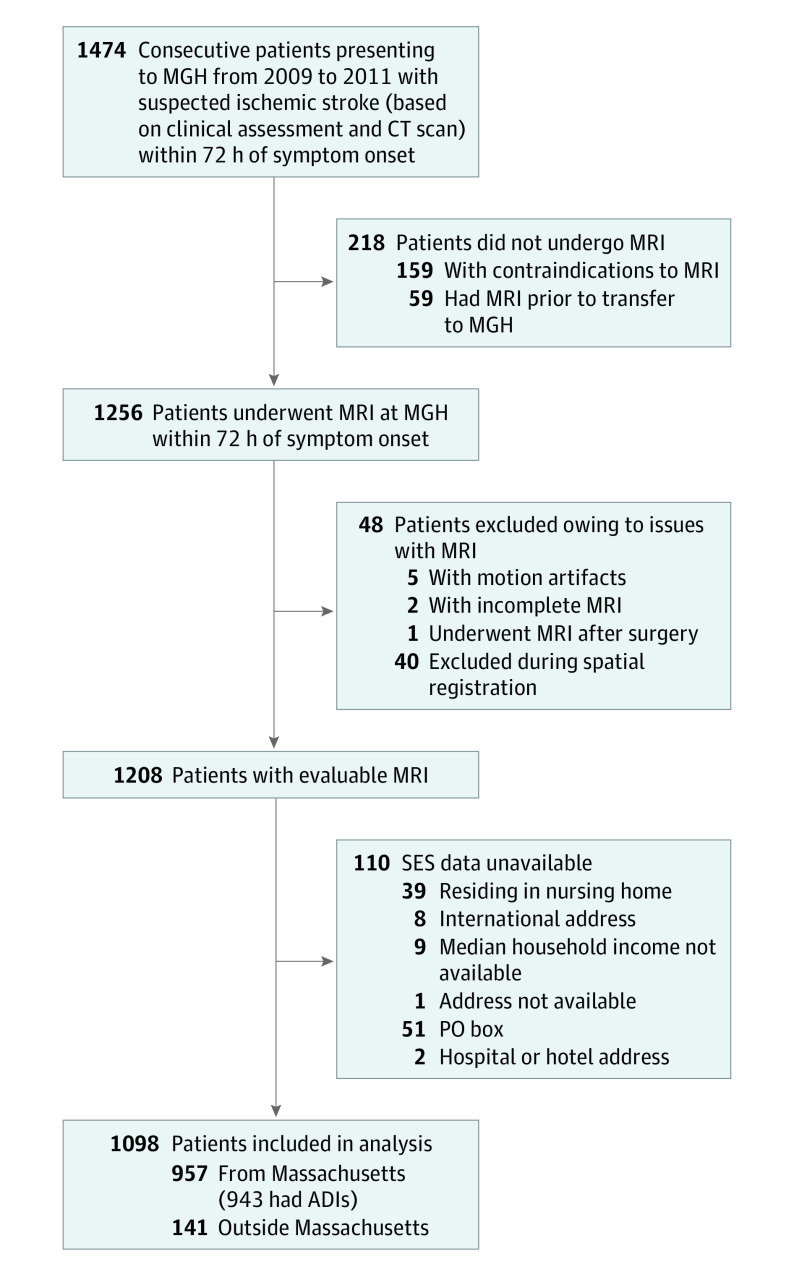
Study Design Flowchart ADI indicates area deprivation index; CT, computed tomography; MGH, Massachusetts General Hospital; MRI, magnetic resonance imaging; PO, post office; and SES, socioeconomic status.

A flowchart of the study design is presented in Figure 1. The study population included 1098 individuals (Table 1) from 338 zip codes (mean [SD] age, 68.1 [15.7] years; 607 men [55.3%] and 491 women [44.7%]). In terms of race and ethnicity, 38 patients (3.5%) were Asian, 55 (5.0%) were Black, 58 (5.3%) were Hispanic, 925 (84.2%) were White, and 22 (2.0%) were other (including American Indian or Alaska Native, more than 1 race or ethnicity, and unknown). The mean (SD) number of patients from any given zip code was 3.0 (5.4). Median household income of the study population ($75 900 [range, $26 100-$191 700) was similar to that of Massachusetts in 2016 ($71 000). There was no difference in income for those who underwent MRI (included in the study) and those who did not (eMethods 6 in the Supplement). For the ADI analyses, 943 patients were derived from 707 Massachusetts census block groups (mean [SD] number of patients per block group, 1.00 [0.65]).

**Table 1.** Patient Characteristics

Characteristic	All population (N = 1098)	Population by income quintile					*P* value^a^
		First (n = 207)	Second (n = 233)	Third (n = 219)	Fourth (n = 220)	Fifth (n = 219)	
Median income, mean (SD) [range], $1000	75.9 (26.7) [26.1-191.7]	45.3 (7.4) [26.1-51.9]	58.1 (4.5) [52.2-63.9]	74.0 (4.8) [63.9-79.6]	85.3 (3.8) [79.7-92.1]	116.4 (22.2) [92.2-191.7]	<.001
Age, mean (SD), y	68.1 (15.7)	67.8 (15.0)	68.1 (16.6)	67.6 (15.6)	67.7 (15.9)	69.1 (15.3)	.85
Race and ethnicity							
Asian	38 (3.5)	7 (3.4)	11 (4.7)	5 (2.3)	8 (3.6)	7 (3.2)	<.001
Black	55 (5.0)	14 (6.8)	21 (9.0)	9 (4.1)	8 (3.6)	3 (1.4)	
Hispanic	58 (5.3)	26 (12.5)	19 (8.1)	5 (2.3)	5 (2.3)	3 (1.4)	
White	925 (84.2)	154 (74.4)	177 (76.0)	196 (89.5)	196 (89.1)	202 (92.2)	
Other^b^	22 (2.0)	6 (2.9)	5 (2.1)	4 (1.8)	3 (1.4)	4 (1.8)	
Sex							
Men	607 (55.3)	114 (55.1)	133 (57.1)	121 (55.3)	107 (48.6)	132 (60.3)	.17
Women	491 (44.7)	93 (44.9)	100 (42.9)	98 (44.7)	113 (51.4)	87 (39.7)	
Hypertension	811 (73.9)	163 (78.7)	171 (73.4)	165 (75.3)	157 (71.4)	155 (70.8)	.33
Diabetes	281 (25.6)	68 (32.9)	68 (29.2)	49 (22.4)	54 (24.5)	42 (19.2)	.01
Hyperlipidemia	530 (48.3)	103 (49.7)	122 (52.4)	103 (47.0)	102 (46.4)	100 (45.7)	.59
Atrial fibrillation	248 (22.6)	41 (19.8)	54 (23.2)	50 (22.8)	49 (22.3)	54 (24.7)	.82
Current smoking	185 (16.9)	45 (21.7)	46 (19.7)	29 (13.2)	36 (16.4)	29 (13.2)	.10
Coronary artery disease	225 (20.5)	42 (20.3)	54 (23.2)	47 (21.5)	36 (16.4)	46 (21.0)	.48
Congestive heart failure	72 (6.5)	14 (6.8)	21 (9.0)	12 (5.5)	13 (5.9)	12 (5.5)	.51
Prior stroke	241 (21.9)	41 (19.8)	56 (24.0)	48 (21.9)	50 (22.7)	46 (21.0)	.86
Baseline mRS score, median (IQR)^c^	0 (0-0)	0 (0-0)	0 (0-0)	0 (0-0)	0 (0-0)	0 (0-0)	.95
Prior TIA	83 (7.5)	15 (7.2)	19 (8.1)	20 (9.1)	13 (5.9)	16 (7.3)	.77
Prestroke medications^d^							
Aspirin	449 (41.2)	96 (46.6)	77 (33.3)	88 (40.4)	93 (42.5)	95 (44.2)	.05
Antiplatelet drugs other than aspirin	86 (7.9)	16 (7.8)	10 (4.3)	21 (9.6)	23 (10.5)	16 (7.4)	.13
Statin	421 (38.7)	86 (41.7)	83 (35.9)	77 (35.3)	97 (44.3)	78 (36.3)	.20
Antihypertensive	663 (60.9)	117 (56.8)	138 (59.7)	133 (61.0)	141 (64.4)	134 (62.3)	.58
Anticoagulant	108 (9.9)	26 (12.6)	24 (10.4)	20 (9.2)	16 (7.3)	22 (10.2)	.47
Insurance	744 (67.7)	137 (66.2)	165 (70.8)	138 (63.0)	156 (70.9)	148 (67.6)	.34
Time from symptom onset to admission, mean (SD), h	12.9 (5.5)	12.8 (5.3)	13.1 (5.9)	13.0 (5.4)	12.8 (5.6)	12.9 (5.4)	.97
Time from admission to MRI, median (IQR), h	9.40 (4.75-21.98)	7.88 (4.56-20.73)	9.33 (4.75-21.51)	8.28 (5.10-20.66)	8.68 (4.17-20.64)	11.36 (5.80-24.61)	.09
Initial clinical severity score, median (IQR)^e^	4 (1-10)	4 (1-12)	5 (2-12)	4 (1-10)	3 (1-9)	1 (1-9)	.009
Infarct volume, median (IQR), mL	6.25 (1.30-26.48)	8.60 (1.50-34.00)	7.50 (1.75-28.45)	6.40 (1.40-30.20)	6.50 (1.13-25.90)	3.60 (0.90-18.40)	.02
Stroke subtypes^f^							
LAA	225 (20.6)	50 (24.5)	42 (18.2)	42 (19.2)	45 (20.5)	46 (21.1)	.60
CE	502 (46.0)	87 (42.6)	100 (43.3)	100 (45.7)	102 (46.6)	113 (51.8)	
SAO	129 (11.8)	24 (11.8)	31 (13.4)	24 (10.9)	24 (10.9)	26 (11.9)	
Other	105 (9.6)	21 (10.3)	24 (10.4)	27 (12.3)	19 (8.7)	14 (6.4)	
Undetermined	130 (11.9)	22 (10.8)	34 (14.7)	26 (11.9)	29 (13.2)	19 (8.7)	
Thrombolytic therapy							
Intravenous	215 (19.6)	50 (24.2)	45 (19.3)	52 (23.7)	40 (18.2)	28 (12.8)	.01
Intra-arterial	52 (4.7)	12 (5.8)	13 (5.6)	10 (4.6)	8 (3.6)	9 (4.1)	.66
Stent placement	4 (0.6)	1 (0.5)	0	2 (0.9)	1 (0.5)	0	.49
Discharge medications							
Aspirin	709 (64.6)	144 (69.6)	136 (58.4)	148 (67.6)	139 (63.2)	142 (64.8)	.12
Antiplatelet drugs other than aspirin	142 (12.9)	32 (15.5)	30 (12.9)	25 (11.4)	32 (14.5)	23 (10.5)	.51
Any antiplatelet	776 (70.7)	159 (76.8)	157 (67.4)	156 (71.2)	152 (69.1)	152 (69.4)	.24
Anticoagulant	409 (37.2)	72 (34.8)	100 (42.9)	75 (34.2)	79 (35.9)	83 (37.9)	.31
Any antiplatelet or anticoagulant^g^	974 (88.7)	183 (88.4)	203 (87.1)	197 (89.9)	196 (89.1)	195 (89.0)	.91
Statin	728 (66.3)	139 (67.1)	147 (63.1)	149 (68.0)	151 (68.6)	142 (64.8)	.70
Antihypertensive	562 (51.2)	111 (53.6)	118 (50.6)	119 (54.3)	111 (50.5)	103 (47.0)	.55
β-Blockers	346 (31.5)	63 (30.4)	76 (32.6)	81 (37.0)	68 (30.9)	58 (26.5)	.20
ACE inhibitors	363 (33.1)	81 (39.3)	80 (34.3)	68 (31.1)	72 (32.7)	62 (28.3)	.17
Functional outcome score, median (IQR)^h^	2 (1-4)	2 (1-4)	2 (1-4)	2 (1-4)	2 (0-3)	1 (0-3)	.001

Abbreviations: ACE, angiotensin-converting enzyme; CE, cardioaortic embolic; LAA, large artery atherosclerosis; MRI, magnetic resonance imaging; mRS, modified Rankin Scale; SAO, small artery occlusion; TIA, transient ischemic attack.

^a^ Calculated across group comparisons.

^b^ Includes American Indian or Alaska Native, more than 1 race or ethnicity, and unknown race or ethnicity.

^c^ Scores range from 0 to 6, with higher scores indicating worse functional outcome.

^d^ Available for 1091 patients.

^e^ Assessed by National Institute of Health Stroke Scale score, ranging from 0 to 42, with higher scores indicating more severe stroke.

^f^ Available for 1091 patients.

^g^ Of the 1017 patients discharged alive, 974 (95.6%) were discharged receiving antiplatelet or anticoagulant drugs.

^h^ Assessed by the mRS score.

The median (IQR) admission NIHSS score was 4 (1-10), which is similar to reported values for the general population with stroke.^31^ The distribution of stroke risk factors was similar across quantiles of median income, except for diabetes, which was more prevalent in the lower quantiles (68 of 207 [32.9%] in the first quintile vs 42 of 219 [19.2%] in the fifth quintile; *P* = .01). Patients in lower SES quantiles were more likely to receive advanced therapies, including intravenous thrombolytic therapy (50 of 207 [24.2%] in the first quintile vs 28 of 219 [12.8%] in the fifth quintile; *P* = .01) (Table 1). Such advanced therapies were more often given to individuals presenting with more severe strokes. There was no difference in time to MRI between SES quintiles (*H*_2_ = 8.09; *P* = .09), and time to MRI was not associated with infarct volume (standardized β, −0.006 [95% CI, −0.161 to 0.129]; *P* = .89). The distribution of stroke locations did not differ across income quintiles (eTable 1 in the Supplement).

### SES vs Initial Stroke Severity

**Figure 2.**
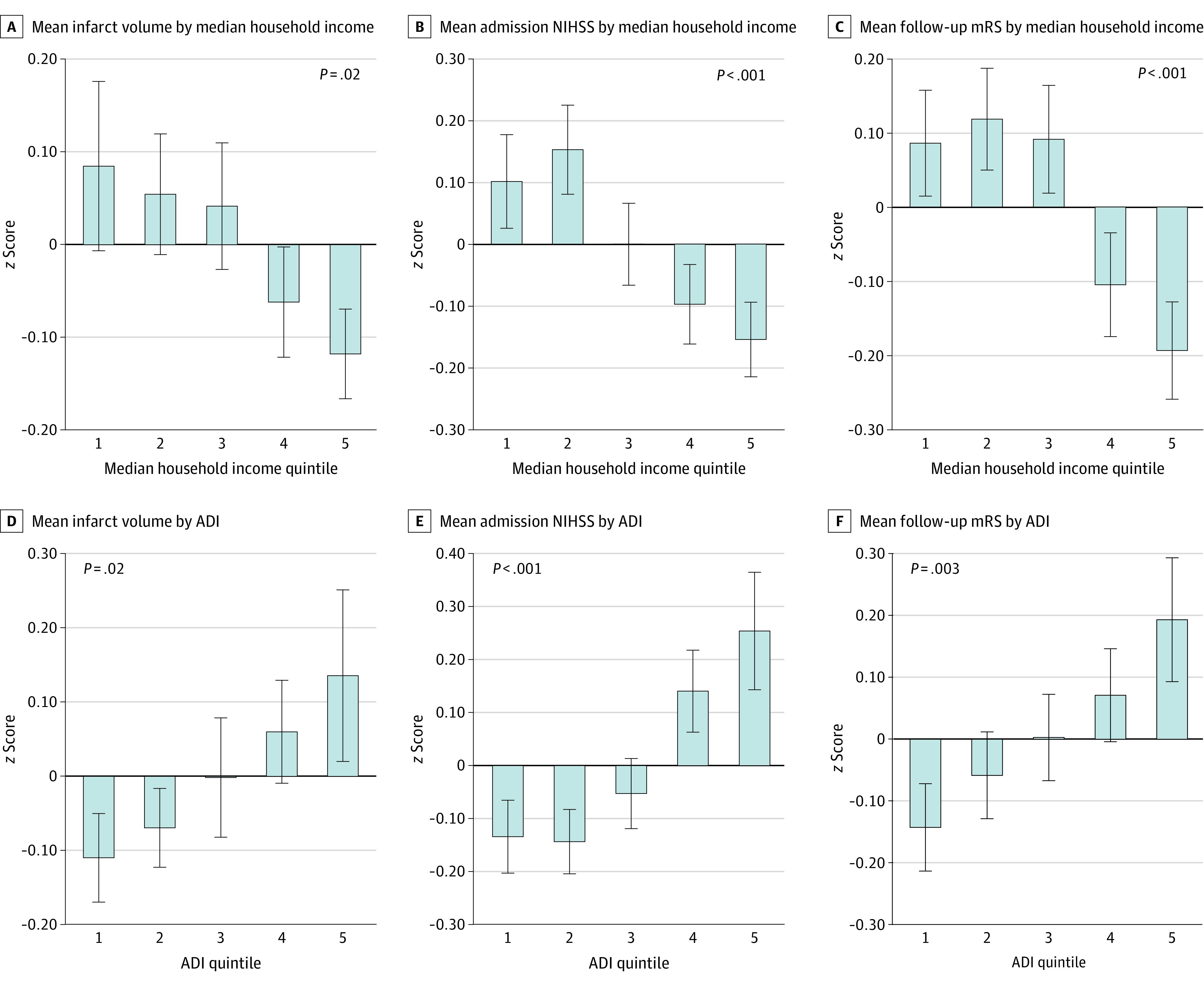
Socieconomic Status Measures vs Study End Points Measures of SES included income (A-C) and area deprivation index (ADI) (D-F) in quintiles. Infarct size was measured using volume on magnetic resonance imaging; clinical stroke severity on admission, using the National Institutes of Health Stroke Scale (NIHSS); and 90-day functional outcome, using the modified Rankin Scale (mRS). Error bars indicate 1 SD.

Lower income was associated with larger infarct volume (standardized β adjusted for stroke risk factors, −0.074 [95% CI, −0.127 to −0.020]; *P* = .007); that is, for every 1-SD decrease in median income, there was a 0.074-SD increase in infarct volume (Table 2, Figure 2A, and eFigure 1 in the Supplement), remaining significant through multivariable adjustments. Alternatively expressed, each $10 000 decrease in income was associated with a 1.67-cm^3^ increase in infarct volume (eTable 2 and eFigure 2 in the Supplement).

**Table 2.** Association Between SES Measures and Initial Stroke Severity Indexes

Model covariable	Median income and initial stroke severity indexes (n = 1098)				ADI and initial stroke severity indexes (n = 943)			
	Infarct volume		NIHSS score		Infarct volume		NIHSS score	
	Standardized β (95% CI)	*P* value	Standardized β (95% CI)	*P* value	Standardized β (95% CI)	*P* value	Standardized β (95% CI)	*P* value
None (unadjusted)	−0.075 (−0.134 to −0.016)	.01	−0.112 (−0.172 to −0.053)	<.001	0.029 (0.005 to 0.053)	.02	0.055 (0.031 to 0.079)	<.001
Age, sex, and race and ethnicity	−0.075 (−0.134 to −0.016)	.01	−0.104 (−0.164 to −0.044)	.001	0.029 (0.005 to 0.053)	.02	0.059 (0.035 to 0.083)	<.001
Stroke risk factors^a^	−0.074 (−0.127 to −0.020)	.007	−0.113 (−0.171 to −0.054)	<.001	0.028 (0.007 to 0.050)	.01	0.056 (0.032 to 0.079)	<.001
Atherosclerotic risk factors^b^	−0.068 (−0.123 to −0.014)	.01	−0.111 (−0.171 to −0.052)	<.001	0.029 (0.007 to 0.051)	.009	0.058 (0.034 to 0.082)	<.001
Prestroke medications^c^	−0.075 (−0.134 to −0.016)	.01	−0.113 (−0.173 to −0.053)	<.001	0.028 (0.004 to 0.052)	.02	0.055 (0.031 to 0.079)	<.001
Time from symptom onset to admission	−0.073 (−0.140 to −0.007)	.03	−0.111 (−0.175 to −0.046)	.001	0.029 (0.002 to 0.056)	.04	0.054 (0.028 to 0.080)	<.001
Health insurance	−0.075 (−0.134 to −0.016)	.01	−0.113 (−0.173 to −0.053)	<.001	0.029 (0.005 to 0.053)	.02	0.055 (0.031 to 0.079)	<.001
All covariables combined	−0.067 (−0.126 to −0.008)	.03	−0.098 (−0.161 to −0.034)	.003	0.027 (0.003 to 0.050)	.03	0.049 (0.023 to 0.075)	<.001

Abbreviations: ADI, area deprivation index; NIHSS, National Institutes of Health Stroke Scale; SES, socioeconomic status.

^a^ Includes age, sex, smoking, diabetes, hypertension, dyslipidemia, history of stroke or TIA, atrial fibrillation, coronary artery disease, and congestive heart failure.

^b^ Includes age, sex, smoking, diabetes, hypertension, and dyslipidemia.

^c^ Includes antiplatelets, statins, and anticoagulants.

Similarly, an inverse association was observed between income and NIHSS on admission (standardized β, −0.113 [95% CI, −0.171 to −0.054]; *P* < .001) (Table 2 and Figure 2B), remaining significant in multivariable analyses. When expressed as nonstandardized variables, each $10 000 decrease in income was associated with a 0.3-point increase in admission NIHSS score (eTable 2 and eFigure 2 in the Supplement).

In addition, higher ADI (ie, greater neighborhood deprivation) was associated with larger infarct volume (standardized β, 0.028 [95% CI, 0.007-0.050]; *P* = .01) and greater NIHSS score (standardized β, 0.056 [95% CI, 0.032-0.079]; *P* < .001). Each decile increase in ADI was associated with a 0.028-SD increase in infarct volume and a 0.055-SD increase in NIHSS score (Table 2, Figure 2D and E, and eFigure 2 in the Supplement).

Although the study population was representative of the general population with stroke, greater health care access for individuals with higher SES may have influenced the findings. Within this scenario, individuals with higher SES and health care access could have a lower threshold to present for care. To exclude this possibility, a subgroup analysis was performed that excluded individuals presenting with minor strokes (ie, NIHSS score ≤4),^32,33^ and lower SES (lowest vs highest quintile) remained associated with greater infarct volume (standardized β, −0.127 [95% CI, −0.182 to −0.073], *P* < .001) and clinical stroke severity (standardized β, −0.257 [95% CI, −0.310 to −0.204]; *P* < .001) (eTable 3 in the Supplement).

### SES vs Long-term Functional Outcome of Stroke

Income was associated with 90-day disability (standardized β, −0.092 [95% CI, −0.149 to −0.035]; *P* = .001) (Figure 2C and eTable 4 and eFigure 2C in the Supplement), remaining significant after adjusting for acute stroke therapies, including thrombolytic therapies and percutaneous revascularization (standardized β, −0.080 [95% CI, −0.147 to −0.012]; *P* = .02). The ADI was also associated with functional outcome (standardized β, 0.038 [95% CI, 0.013-0.062]; *P* = .001) (Figure 2F and eTable 4 and eFigure 2F in the Supplement). Accounting for various stroke locations yielded similar results (eTable 5 in the Supplement). Similarly significant results were obtained using ordinal regression (eTable 6 in the Supplement). Similarly, stroke severity on presentation (both infarct volume and NIHSS score) was associated with 90-day disability (eTable 7 in the Supplement).

To further test for potential confounding effects of therapies, we conducted a subgroup analysis of patients who did not receive reperfusion therapies. In this group, both median income (standardized β, −0.076 [95% CI, −0.138 to −0.015]; *P* = .005) and ADI (standardized β, 0.033 [95% CI, 0.008-0.059]; *P* = .01) remained associated with functional outcome (eTable 8 in the Supplement).

### Mediation Analysis

Mediation analyses were performed to explore putative mechanisms underlying the association between SES and long-term disability. First, single-mediator analyses were performed to test whether infarct volume significantly mediated the association between SES and NIHSS score (eFigure 3 in the Supplement). The pathways of decreased income to increased infarct volume to increased NIHSS score (standardized β, −0.039 [95% CI, −0.066 to −0.013]; *P* < .05) and increased ADI to increased infarct volume to increased NIHSS score (standardized β, 0.033 [95% CI, 0.007-0.064]; *P* < .05) were significant.

**Figure 3.**
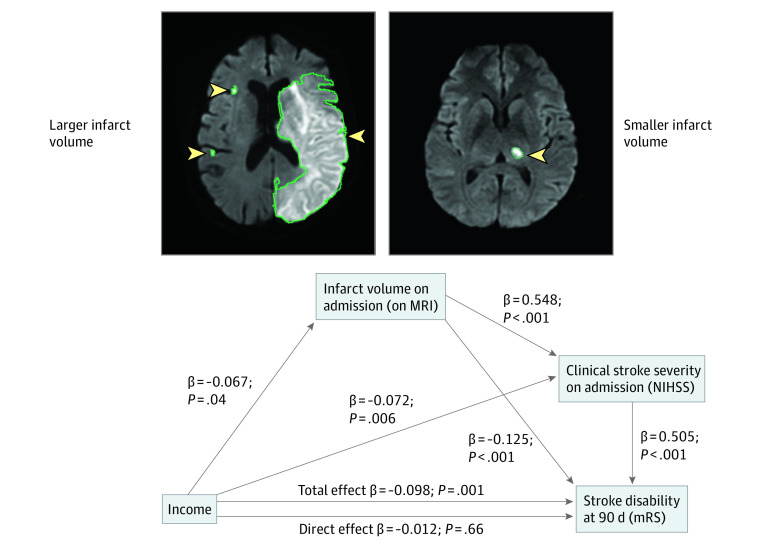
Multistep Path Linking Socioeconomic Status to Functional Outcome After Stroke A serial 2-mediator model shows that initial stroke severity indexes significantly mediate the association between income and functional outcome (modified Rankin Scale [mRS]). Within this model, all indirect pathways involving both infarct volume or admission National Institutes of Health Stroke Scale (NIHSS) score (whether alone or in series) were statistically significant. The direct path (which excludes a role for initial stroke severity indexes) becomes nonsignificant. Regression coefficients and *P* values correspond to models adjusted for age and sex. Arrowheads and green lines indicate the location and border of infarct, respectively. MRI indicates magnetic resonance imaging. The following 3 distinct indirect pathways were associated with 90-day disability: (1) decreased income to increased infarct volume to increased 90-day disability (standardized β, −0.008 [95% CI, −0.016 to −0.004]; *P* < .05); (2) decreased income to increased infarct volume to increased NIHSS score to increased 90-day disability (standardized β, −0.018 [95% CI, −0.032 to −0.004]; *P* < .05); and (3) decreased income to increased NIHSS score to increased 90-day disability (standardized β, −0.036 [95% CI, −0.061 to −0.012]; *P* < .05). Collectively these 3 indirect pathways account for 64% of the association between socioeconomic status and long-term disability (standardized β, −0.063 [95% CI, −0.095 to −0.029]; *P* < .05).

Next, dual-mediator models were evaluated using income as the predictive factor, infarct volume and NIHSS score as mediators, and modified Rankin Scale score as the outcome (Figure 3). In these analyses, 3 distinct pathways were associated with 90-day disability:

1. decreased income to increased infarct volume to increased 90-day disability (standardized β, −0.008 [95% CI, −0.016 to −0.004]; *P* < .05);
2. decreased income to increased infarct volume to increased NIHSS score to increased 90-day disability (standardized β, −0.018 [95% CI, −0.032 to −0.004]; *P* < .05); and
3. decreased income to increased NIHSS score to increased 90-day disability (standardized β, −0.036 [95% CI, −0.061 to −0.012]; *P* < .05).

Collectively, these 3 indirect paths indicate that initial stroke severity (measured as infarct volume or NIHSS score) accounts for 64% of the association between SES and long-term disability (standardized β, −0.063 [95% CI, −0.095 to −0.029]; *P* < .05) in an age- and sex-adjusted model. With further multivariable adjustments, these paths account for 64% to 77% of the association between SES and functional outcome (eTable 9 in the Supplement). Similar findings were observed when ADI was used to evaluate SES (eFigure 4 in the Supplement).

### Etiologic Stroke Subtypes

Associations were observed between SES and infarct volume among individuals presenting with LAA and CE strokes, but not other stroke subtypes, suggesting that the association between SES and initial stroke severity was driven by individuals presenting with these 2 most common subtypes (eTables 9 and 10 and eFigure 5 in the Supplement). Among individuals with LAA or CE stroke subtypes, income was inversely associated with infarct volume (standardized β, −0.089 [95% CI, −0.157 to −0.022]; *P* = .009) and NIHSS score (standardized β, −0.156 [95% CI, −0.230 to −0.083]; *P* < .001). Similarly, a higher ADI was associated with infarct volume (standardized β, 0.038 [95% CI, 0.011-0.065]; *P* = .006) and NIHSS score (standardized β, 0.075 [95% CI, 0.045-0.106]; *P* < .001). Importantly, the distribution of stroke subtypes did not vary by income (Table 1).

Furthermore, both income and ADI were associated with 90-day disability among patients presenting with LAA (standardized β, −0.115 [95% CI, −0.184 to −0.045]; *P* = .001) and CE (standardized β, 0.042 [95% CI, 0.013-0.071]; *P* = .005) stroke subtypes (eTable 11 in the Supplement). No association was seen between SES and initial stroke severity or functional outcome for other stroke subtypes.

## Discussion

This study found that lower SES was independently associated with more severe strokes on presentation, manifested by larger infarct volumes and greater initial clinical severity. These larger initial stroke measures were associated with worse long-term functional outcomes, independently of prestroke risk factors and treatments, symptom duration, and poststroke treatments. Moreover, most of the observed association between SES and long-term functional outcomes was explained by SES-related differences in initial stroke severity. Further, the SES-related disparities in stroke size appeared to be associated with large-artery atherosclerosis and cardioembolic mechanisms. Collectively, these findings suggest that SES-related disparities in stroke outcomes (1) are independent of clinical confounders, (2) are manifest before care delivery, and (3) may involve biological factors.

### Association Between SES and Initial Stroke Severity

To evaluate the association of SES-related disparities with stroke outcomes, we leveraged a single-center design at a high-volume stroke center with widely available advanced imaging and acute stroke service care. Individuals with lower SES were more likely to receive acute reperfusion therapies and were equally likely to undergo MRI. In other words, strokes among those with lower SES were more severe and required more advanced treatments. Nonetheless, lower SES was associated with larger infarcts and greater 90-day disability after accounting for prestroke and poststroke therapies and other confounders.

The use of imaging to evaluate the association between SES and stroke functional outcomes was a strength of this study. Prior studies^6,7,8,10^ relied solely on clinical scales of initial stroke severity. Moreover, neither initial clinical severity on its own nor conventional risk factors have fully explained this association.^8,34^ This prompted the conclusion that much of the association between SES and long-term stroke outcomes is driven by differences in poststroke care, such as inadequate access to health care and rehabilitation services in groups with lower SES.^5^ The present results from a single tertiary care center show that stroke severity at presentation (assessed by both infarct volume and NIHSS score) explained 64% of the association between SES and functional outcome. These findings further shift culpability for SES-related differences in long-term stroke outcomes from poststroke factors to those that precede presentation. More effort should be directed toward stroke prevention in this vulnerable group, and future research should address the presence of nontraditional risk factors that contribute to larger and more severe strokes in this group.

This study also provides clues to the mechanism underlying SES-related disparities. First, the associations between SES and infarct size and severity remained unattenuated after accounting for prestroke treatments or risk factors, insurance status, and race and ethnicity, suggesting that these factors alone do not explain the association. Second, SES-associated disparities in infarct size and stroke severity were mainly observed within the LAA and CE subtypes; however, atherosclerosis risk factors and cardioemboli alone do not explain this association. Accordingly, these findings suggest a biological link between SES and stroke beyond differences in risk factors alone.

Inflammation represents a potential mechanism that may explain this biological link. In nonhuman primates, lower social status leads to increased inflammation.^35^ Human studies have yielded similar findings.^36^ The mechanism linking SES with inflammation may involve stress-associated neurobiological mechanisms.^37,38,39^ Although heightened inflammation increases the likelihood of strokes,^40^ it may also worsen stroke severity. For example, inflammation can lead to larger thrombi and emboli,^41,42^ which could occlude vessels more proximally and cause larger infarctions.^43^ Future studies should be designed to directly investigate this hypothesis.

### Strengths and Limitations

Strengths of the present study include the large number of consecutive patients enrolled, the prospective single-center design, and the consistent availability of advanced care. Moreover, we accounted for a number of potential confounders, some of which have not been previously assessed in similar studies.

Our study is not without limitations. Data on duration of residence at the provided address for each patient were not available. Individual level SES data were not available, and they were derived at the zip code and Census block group levels. Nevertheless, although zip code−level SES measures are less precise than individual SES, they are associated with adverse health outcomes.^39,44,45,46^ Importantly, because a given zip code may be heterogeneous with respect to disease and socioeconomic indicators,^47^ we leveraged ADI in secondary analyses.^24^ The fact that 2 distinct and validated SES metrics provided similar results reinforces the findings.

Several studies^48^ suggest that low SES adversely affects medication adherence, which could not be assessed in this study. Patients with milder strokes in the lower SES group may have been less likely to seek care owing to cost, which could have led to an imbalance of stroke severity between SES groups. Nevertheless, this seems unlikely, because before the study’s enrollment period, Massachusetts mandated and provided health insurance for all residents. It also seems unlikely that individuals with lower SES who develop minor strokes (ie, NIHSS score ≤4) might be less likely to seek care than those of higher SES, given that there is a similar median NIHSS score to that of the general population with stroke^31^ and that analyses excluding patients with NIHSS scores of 4 or less still showed an association between SES and initial stroke severity.

Magnetic resonance imaging was performed within 72 hours of symptom onset but not necessarily at presentation to avoid unnecessary treatment delays. Thus, in 232 patients, MRI was completed after reperfusion therapies, which may have affected infarct volume in this subgroup. Importantly, this limitation is unlikely to affect our results, because (1) time to MRI did not differ between SES groups (and was not associated with infarct size), and (2) sensitivity analyses excluding patients who underwent imaging after reperfusion therapies yielded similar results.

Our study cohort included interhospital transfers; however, the data describing which patients were admitted as transfers and which were not ultimately transferred were not available, potentially introducing a degree of selection bias. Other factors that were not evaluated in this study may contribute to the association between SES and stroke severity, including air pollution^49,50^ and health behaviors (eg, diet, exercise). Last, the single-center design and lack of racial and ethnic diversity of the population may limit its generalizability.

## Conclusions

In this single-center cohort study using quantitative imaging, we observed independent associations between socioeconomic status and infarct volume as well as poststroke disability. The major association of SES with disability appears to be a consequence of infarct volume on presentation and is not explained by confounding factors. More research is needed to further uncover the mechanisms leading to these observations with the aim of reducing the burden of disability in groups with lower SES.

## Notes

Supplement. **eMethods 1.** Area Deprivation Index **eMethods 2.** Infarct Volume Measurement by MRI **eMethods 3.** The Causative Classification System of Stroke (CCS) **eMethods 4.** Assumptions of Linear Regression **eMethods 5.** Mediation Analysis **eMethods 6.** Income for Patients Who Underwent MRI (Included in the Study) and Those Who Did Not (Excluded) **eTable 1.** Distribution of Different Stroke Locations Across Income Quintiles **eTable 2.** Association Between SES Measures and Initial Stroke Severity Indices (Using Income in $10 000 Increments, Infarct Volume in Cubic Centimeters, and NIHSS in Point Form) **eTable 3.** Association Between Extremes of Median Income (First and Fifth Quintiles) and Initial Stroke Severity Indices After Excluding Patients With Minor Strokes (NIHSS Score ≤4) **eTable 4.** Association Between SES Measures and Functional Outcome at 90 Days **eTable 5.** Association Between SES Measures and Functional Outcome at 90 Days Adjusted for Stroke Location **eTable 6.** Association Between SES Measures and Functional Outcome at 90 Days Using Ordinal Regression Model **eTable 7.** Association Between Initial Stroke Severity Indices and Functional Outcome at 90 Days **eTable 8.** Association Between SES Measures and Functional Outcome at 90 Days Within the Subset of Patients Who Did Not Receive Reperfusion Therapies **eTable 9.** Mediation Analysis Models With Multivariable Adjustments **eTable 10.** Association Between SES Measures and Initial Stroke Severity Indices (Within the Subgroup of Patients With LAA and CE Strokes) **eTable 11.** Association Between SES Measures and Functional Outcome at 90 Days (Within the Subgroup of Patients With LAA and CE Strokes) **eFigure 1.** Distribution of Infarct Volume by Median Household Income **eFigure 2.** Association Between Different SES Measures (Income and ADI) and Study End Points **eFigure 3.** Single-Mediator Mediation Analyses **eFigure 4.** A Serial 2-Mediator Mediation Analysis **eFigure 5.** Association Between Different SES Measures (Income and ADI) vs Study End Points Within the Subgroup of Patients With LAA and CE Strokes
eReferences
