# Supplementary material for: Association of Socioeconomic Status and Infarct Volume With Functional Outcome in Patients With Ischemic Stroke
Source: JAMA Netw Open. 2022 Apr 27;5(4):e229178. doi: 10.1001/jamanetworkopen.2022.9178 (PMC9047646; doi:10.1001/jamanetworkopen.2022.9178)
Supplement: Supplement. — eMethods 1. Area Deprivation Index eMethods 2. Infarct Volume Measurement by MRI eMethods 3. The Causative Classification System of Stroke (CCS) eMethods 4. Assumptions of Linear Regression eMethods 5. Mediation Analysis eMethods 6. Income for Patients Who Underwent MRI (Included in the Study) and Those Who Did Not (Excluded) eTable 1. Distribution of Different Stroke Locations Across Income Quintiles eTable 2. Association Between SES Measures and Initial Stroke Severity Indices (Using Income in $10 000 Increments, Infarct Volume in Cubic Centimeters, and NIHSS in Point Form) eTable 3. Association Between Extremes of Median Income (First and Fifth Quintiles) and Initial Stroke Severity Indices After Excluding Patients With Minor Strokes (NIHSS Score ≤4) eTable 4. Association Between SES Measures and Functional Outcome at 90 Days eTable 5. Association Between SES Measures and Functional Outcome at 90 Days Adjusted for Stroke Location eTable 6. Association Between SES Measures and Functional Outcome at 90 Days Using Ordinal Regression Model eTable 7. Association Between Initial Stroke Severity Indices and Functional Outcome at 90 Days eTable 8. Association Between SES Measures and Functional Outcome at 90 Days Within the Subset of Patients Who Did Not Receive Reperfusion Therapies eTable 9. Mediation Analysis Models With Multivariable Adjustments eTable 10. Association Between SES Measures and Initial Stroke Severity Indices (Within the Subgroup of Patients With LAA and CE Strokes) eTable 11. Association Between SES Measures and Functional Outcome at 90 Days (Within the Subgroup of Patients With LAA and CE Strokes) eFigure 1. Distribution of Infarct Volume by Median Household Income eFigure 2. Association Between Different SES Measures (Income and ADI) and Study End Points eFigure 3. Single-Mediator Mediation Analyses eFigure 4. A Serial 2-Mediator Mediation Analysis eFigure 5. Association Between Different SES Measures (Income and ADI) vs Study End Points Within the [file jamanetwopen-e229178-s001.pdf]

Supplementary Online Content

Ghoneem A, Osborne MT, Abohashem S, et al. Association of socioeconomic status and infarct volume with functional outcome in patients with ischemic stroke. *JAMA Netw Open*. 2022;5(4):e229178. doi:10.1001/jamanetworkopen.2022.9178

eMethods 1. Area Deprivation Index
eMethods 2. Infarct Volume Measurement by MRI
eMethods 3. The Causative Classification System of Stroke (CCS)
eMethods 4. Assumptions of Linear Regression
eMethods 5. Mediation Analysis
eMethods 6. Income for Patients Who Underwent MRI (Included in the Study) and Those Who Did Not (Excluded)
eTable 1. Distribution of Different Stroke Locations Across Income Quintiles
eTable 2. Association Between SES Measures and Initial Stroke Severity Indices (Using Income in $10 000 Increments, Infarct Volume in Cubic Centimeters, and NIHSS in Point Form)
eTable 3. Association Between Extremes of Median Income (First and Fifth Quintiles) and Initial Stroke Severity Indices After Excluding Patients With Minor Strokes (NIHSS Score ≤4)
eTable 4. Association Between SES Measures and Functional Outcome at 90 Days
eTable 5. Association Between SES Measures and Functional Outcome at 90 Days Adjusted for Stroke Location
eTable 6. Association Between SES Measures and Functional Outcome at 90 Days Using Ordinal Regression Model
eTable 7. Association Between Initial Stroke Severity Indices and Functional Outcome at 90 Days
eTable 8. Association Between SES Measures and Functional Outcome at 90 Days Within the Subset of Patients Who Did Not Receive Reperfusion Therapies
eTable 9. Mediation Analysis Models With Multivariable Adjustments
eTable 10. Association Between SES Measures and Initial Stroke Severity Indices (Within the Subgroup of Patients With LAA and CE Strokes)
eTable 11. Association Between SES Measures and Functional Outcome at 90 Days (Within the Subgroup of Patients With LAA and CE Strokes)
eFigure 1. Distribution of Infarct Volume by Median Household Income
eFigure 2. Association Between Different SES Measures (Income and ADI) and Study End Points
eFigure 3. Single-Mediator Mediation Analyses
eFigure 4. A Serial 2-Mediator Mediation Analysis
eFigure 5. Association Between Different SES Measures (Income and ADI) vs Study End Points Within the Subgroup of Patients With LAA and CE Strokes
eReferences

This supplementary material has been provided by the authors to give readers additional information about their work.

eMethods 1. Area Deprivation Index
ADI combines 17 weighted census indicators (e.g., measures of education, employment, housing quality, poverty).[1]. ADI was collected for all Massachusetts residents in the study population. Each patient address was geo-linked to their Area Deprivation Index (ADI) neighborhood ranking using the mapping function freely available through the Neighborhood Atlas (https://www.neighborhoodatlas.medicine.wisc.edu/).[2] ADI data is available in rank form as deciles within each state with the first decile being least disadvantaged, and the tenth decile being the most disadvantaged.

eMethods 2. Infarct Volume Measurement by MRI
We used a commercially available image display and analysis program (MRIcron). Clinically relevant regions characterized by increased signal intensity on DWI and decreased intensity on the apparent diffusion coefficient maps were classified as acute infarct. Examiners were provided with a brief clinical history and neurological examination findings for each patient. The outlining technique was manual. Lesion volumes were automatically produced by the software based on the slice thickness and overall outlined lesion area. In patients with multiple infarcts, the sum of all infarct volumes was calculated. This technique has excellent interrater reliability with intraclass correlation coefficient ranging between 0.93 and 0.99.[3]

eMethods 3. The Causative Classification System of Stroke (CCS)
The Causative Classification System of Stroke (CCS) is a computerized, evidence-based algorithm that was developed in 2005 and has been used by several stroke centers since then.[4-6] It classifies stroke etiology with high inter-rater agreement rates ranging from 80% to 95%.[7] It was validated against the assessment of 20 expert neurologists with a κ value for causative classification of 0.80 (95% confidence interval [CI] 0.78–0.81) for the 5-subtype CCS.[8] The primary reference was added to the manuscript.

eMethods 4. Assumptions of Linear Regression
To ensure that there was no multicollinearity, a Pearson correlation coefficient was calculated to examine the relationship between predictors. All predictors provided coefficient values closer to (r= 0) rather than (r=0.7 or r= -0.7), tolerance values above 0.1 and variance inflation factor below 10, which in turn suggested that the assumption of multicollinearity wasn't violated. A Durbin-Watson statistic was calculated to assess the assumption that the values of the residuals are independent, which suggested that this assumption wasn't violated (Durbin-Watson=1.807). Cook's Distance values were calculated to ensure that no influential cases were biasing the model. All values were below 1 (maximum Cook's Distance value =0.049), suggesting that no cases were biasing the model.

eMethods 5. Mediation Analysis
Mediation analysis was employed using the SPSS PROCESS macro to evaluate mechanisms underlying the association between SES and long-term disability. The analysis uses an ordinary least squares or logistic regression-based path structure to

© 2022 Ghoneem A et al. *JAMA Network Open*.

estimate direct and indirect effects and confidence intervals (Cis) based upon 5,000 bias-corrected bootstrap samples.[9,10] CIs for multi-step indirect pathways assessed using bootstrapping are frequently asymmetric, which hinders the ability to apply a parametric asymptotic distribution to quantify P values. Accordingly, 95% CIs that do not cross zero are deemed significant with P<0.05.

eMethods 6. Income for Patients Who Underwent MRI (Included in the Study) and Those Who Did Not (Excluded)

Out of 1474 consecutive patients enrolled in our study, 218 patients did not undergo MRI and 48 patients were excluded for technical issues with their scans, yielding a total of 266 patients. Of this group, only 245 of those patients had available income data. The mean (SD) income of these patients was $75463.6 ($25158.4) vs. $75937.1 ($26653.8) for the 1098 patients included in the study (P=0.98).

© 2022 Ghoneem A et al. *JAMA Network Open*.

eTable 1. Distribution of Different Stroke Locations Across Income Quintiles

Characteristic, N (%)	Complete Population	Income quintiles					*P* [b]
		**First (N= 203)**	**Second (N=231)**	**Third (N=219)**	**Fourth (N=219)**	**Fifth (N=218)**	
Stroke Location: Vascular territory (N=1090)							
ICA	53 (4.9)	15 (28.3)	10 (18.9)	15 (28.3)	10 (18.9)	3 (5.7)	0.17
MCA	583 (53.5)	105 (18.0)	116 (19.9)	114 (19.6)	122 (20.9)	126 (21.6)	
ACA	13 (1.2)	4 (30.8)	2 (15.4)	4 (30.8)	2 (15.4)	1 (7.7)	
PCA	89 (8.2)	16 (18.0)	18 (20.2)	17 (19.1)	21 (23.6)	17 (19.1)	
Cerebellar	46 (4.2)	11 (23.9)	7 (15.2)	10 (21.7)	3 (6.5)	15 (32.6)	
Brainstem	73 (6.7)	14 (19.2)	19 (26.0)	11 (15.1)	13 (17.8)	16 (21.9)	
Multiple	233 (21.4)	38 (16.3)	59 (25.3)	48 (20.6)	48 (20.6)	40 (17.2)	
Stroke Location: anterior, posterior or both (N=1089)							0.96
Anterior	734 (67.4)	137 (18.7)	152 (20.7)	152 (20.7)	150 (20.4)	143 (19.5)	
Posterior	258 (23.7)	50 (19.4)	55 (21.3)	46 (17.8)	51 (19.8)	56 (21.7)	
Both	97 (8.9)	16 (16.5)	24 (24.7)	21 (21.6)	18 (18.6)	18 (18.6)	
Stroke Location: laterality (N=1090)							
Left	485 (44.5)	99 (20.4)	93 (19.2)	93 (19.2)	104 (21.4)	96 (19.8)	0.628
Right	414 (38)	74 (17.9)	90 (21.7)	87 (21)	76 (18.4)	87 (21)	
Both	191 (17.5)	30 (15.7)	48 (25.1)	39 (20.4)	39 (20.4)	35 (18.3)	

© 2022 Ghoneem A et al. *JAMA Network Open*.

eTable 2. Association Between SES Measures and Initial Stroke Severity Indices (Using Income in $10 000 Increments, Infarct Volume in Cubic Centimeters, and NIHSS in Point Form)

Models with covariables	Median income and initial stroke severity indices (N=1098)				ADI and initial stroke severity indices (N= 943)			
	Infarct Volume (cc)		NIHSS		Infarct Volume (cc)		NIHSS	
	β (95% CI)	*p*	β (95% CI)	*p*	β (95% CI)	*p*	β (95% CI)	*p*
**NONE** (unadjusted)	-1.700 (-3.308, -0.362)	0.01	-0.299 (-0.457, -0.141)	<0.001	1.726 (0.276, 3.176)	0.02	0.386 (0.215, 0.556)	<0.001
**Age, sex, and race**	-1.700 (-3.308, -0.362)	0.01	-0.273 (-0.431, -0.115)	0.001	1.726 (0.276, 3.176)	0.02	0.412 (0.244, 0.581)	<0.001
**Stroke risk factors** [a]	-1.665 (-2.878, -0.451)	0.007	-0.297 (-0.451, -0.143)	<0.001	1.700 (0.410, 2.991)	0.01	0.391 (0.225, 0.557)	<0.001
**Atherosclerotic risk factors** [b]	-1.545 (-2.776, -0.314)	0.01	-0.294 (-0.450, -0.137)	<0.001	1.735 (0.432, 3.039)	0.009	0.405 (0.236, 0.574)	<0.001
**Pre-stroke medications** [c]	-1.665 (-3.043, -0.356)	0.013	-0.298 (-0.456, -0.140)	<0.001	1.704 (0.246, 3.162)	0.02	0.386 (0.215, 0.558)	<0.001
**Time from symptom onset to admission**	-1.663 (-3.162, -0.164)	0.03	-0.292 (-0.463, -0.120)	<0.001	1.737 (0.081, 3.392)	0.04	0.381 (0.196, 0.566)	<0.001
**Health Insurance**	-1.701 (-3.040, -0.362)	0.01	-0.298 (-0.456, -0.141)	<0.001	1.726 (0.275, 3.176)	0.02	0.385 (0.215, 0.555)	<0.001

© 2022 Ghoneem A et al. *JAMA Network Open*.

**All covariables combined**	-1.516 (-2.850, -0.181)	0.03	-0.258 (-0.425, -0.091)	0.003	1.605 (0.169, 3.041)	0.03	0.347 (0.164, 0.530)	<0.001

a: Age, sex, smoking, diabetes, hypertension, dyslipidemia, history of stroke or TIA, atrial fibrillation, coronary artery disease, and congestive heart failure.
B: Age, sex, smoking, diabetes, hypertension, and dyslipidemia.
C: Antiplatelets, statins, and anticoagulants.

NIHSS: National Institute of Health Stroke Scale**,** ADI: Area Deprivation Index

© 2022 Ghoneem A et al. *JAMA Network Open*.

eTable 3. Association Between Extremes of Median Income (First and Fifth Quintiles) and Initial Stroke Severity Indices After Excluding Patients With Minor Strokes (NIHSS Score ≤4)

**Model covariables**	**Median income and initial stroke severity indices (N= 488)**			
	**Infarct Volume**		**NIHSS**	
	**Standardized β (95% CI)**	***p***	**Standardized β (95% CI)**	***p***
**NONE** (unadjusted)	-0.164 (-0.234, -0.095)	<0.001	-0.285 (-0.338, -0.231)	<0.001
**Age, sex, and race**	-0.164 (-0.234, -0.095)	<0.001	-0.285 (-0.338, -0.231)	<0.001
**Stroke risk factors** [a]	-0.127 (-0.182, -0.073)	<0.001	-0.257 (-0.310, -0.204)	<0.001
**Atherosclerotic risk factors** [b]	-0.127 (-0.182, -0.073)	<0.001	-0.263 (-0.318, -0.209)	<0.001
**Pre-stroke medications** [c]	-0.162 (-0.231, -0.092)	<0.001	-0.284 (-0.338, -0.230)	<0.001
**Time from symptom onset to admission**	-0.155 (-0.234, -0.076)	<0.001	-0.265 (-0.323, -0.207)	<0.001
**Health Insurance**	-0.164 (-0.233, -0.095)	<0.001	-0.284 (-0.337, -0.231)	<0.001
**All covariables combined**	-0.100 (-0.158, -0.041)	0.001	-0.225 (-0.281, -0.170)	<0.001

a: Age, sex, smoking, diabetes, hypertension, dyslipidemia, history of stroke or TIA, atrial fibrillation, coronary artery disease, and congestive heart failure.
b: Age, sex, smoking, diabetes, hypertension, and dyslipidemia.
c: Antiplatelets, statins, and anticoagulants.

NIHSS: National Institute of Health Stroke Scale

© 2022 Ghoneem A et al. *JAMA Network Open*.

eTable 4. Association Between SES Measures and Functional Outcome at 90 Days

Models with covariables	Median income and mRS at 90 days (N=1015)		ADI and mRS at 90 days (N=874)	
	Standardized β (95% CI)	*p*	Standardized β (95% CI)	*p*
**NONE** (unadjusted)	-0.106 (-0.167, -0.045)	0.001	0.039 (0.014, 0.064)	0.002
**Age, sex, and race**	-0.1 (-0.159, -0.042)	0.001	0.047 (0.023, 0.071)	<0.001
**Stroke risk factors** [a]	-0.092 (-0.149, -0.035)	0.001	0.038 (0.015, 0.062)	0.001
**Atherosclerotic risk factors** [b]	-0.092 (-0.150, -0.034)	0.002	0.04 (0.016, 0.064)	0.001
**Pre-stroke medications** [c]	-0.102 (-0.164, -0.041)	0.001	0.038 (0.013, 0.063)	0.003
**Time from symptom onset to admission**	-0.099 (-0.167, -0.031)	0.004	0.036 (0.008, 0.064)	0.01
**Baseline mRS**	-0.099 (-0.158, -0.040)	0.001	0.039 (0.014, 0.063)	0.002
**Insurance**	-0.106 (-0.167, -0.045)	0.001	0.039 (0.014, 0.064)	0.002
**Combined pre-admission variables** [d]	-0.077 (-0.139, -0.015)	0.01	0.036 (0.011, 0.061)	0.005
**Reperfusion therapies** [e]	-0.080 (-0.147, -0.012)	0.02	0.04 (0.013, 0.068)	0.005
**Discharge medications** [f]	-0.106 (-0.165, -0.047)	<0.001	0.040 (0.016, 0.064)	<0.001
**Combined post-admission variables** [g]	-0.069 (-0.133, -0.006)	0.03	0.033 (0.007, 0.059)	0.01

a: Age, sex, smoking, diabetes, hypertension, dyslipidemia, history of stroke or TIA, atrial fibrillation, coronary artery disease, and congestive heart failure.
b: Age, sex, smoking, diabetes, hypertension, and dyslipidemia.
c: Antiplatelets, statins, and anticoagulants.
d: Age, sex, race, stroke risk factors, pre-stroke medications, time from symptom onset to admission, baseline mRS, and insurance.
e: Intravenous/intraarterial thrombolytics and stents.
f: Antiplatelets, statins, antihypertensives, anticoagulants, beta blockers, and ACEI's/ARB's.
g: Reperfusion therapies, discharge medications, and insurance.

NIHSS: National Institute of Health Stroke Scale, mRS: modified Rankin Scale
ADI: Area Deprivation Index

© 2022 Ghoneem A et al. *JAMA Network Open*.

eTable 5. Association Between SES Measures and Functional Outcome at 90 Days Adjusted for Stroke Location

Models with covariables	Median income and mRS at 90 days (N=1007)		ADI vs mRS at 90 days (N=867)	
	Standardized β (95% CI)	*p*	Standardized β (95% CI)	*p*
**Location: vascular territory (ICA, MCA, ACA, PCA, Cerebellar, Brain stem, multiple)**	-0.111 (-0.172, -0.050)	<0.001	0.039 (0.014, 0.064)	0.002
**Location: anterior, posterior or both**	-0.111 (-0.172, -0.049)	<0.001	0.039 (0.014, 0.064)	0.002
**Location: left, right or both**	-0.115 (-0.175, -0.054)	<0.001	0.043 (0.018, 0.068)	<0.001

ICA: Internal Carotid Artery
MCA: Middle Cerebral Artery
ACA: Anterior Cerebral Artery
PCA: Posterior Cerebral Artery

© 2022 Ghoneem A et al. *JAMA Network Open*.

eTable 6. Association Between SES Measures and Functional Outcome at 90 Days Using Ordinal Regression Model

Models with covariables	Ordinal Regression Analysis			
	Median income and mRS at 90 days (N=1015)		ADI and mRS at 90 days (N=874)	
	Standardized β (95% CI)	*p*	Standardized β (95% CI)	*p*
**NONE** (unadjusted)	-0.194 (-0.303, -0.084)	0.001	0.074 (0.029, 0.118)	0.001
**Age, sex, and race**	-0.202 (-0.313, -0.091)	<0.001	0.083 (0.037, 0.129)	<0.001
**Stroke risk factors** [a]	-0.192 (-0.304, -0.079)	0.001	0.074 (0.027, 0.121)	0.002
**Atherosclerotic risk factors** [b]	-0.183 (-0.295, -0.072)	0.001	0.080 (0.035, 0.126)	0.001
**Pre-stroke medications** [c]	-0.192 (-0.301, -0.082)	0.001	0.072 (0.027, 0.117)	0.002
**Time from symptom onset to admission**	-0.177 (-0.298, -0.055)	0.004	0.069 (0.019, 0.119)	0.007
**Baseline mRS**	-0.193 (-0.303, -0.083)	0.001	0.083 (0.038, 0.127)	<0.001
**Insurance**	-0.195 (-0.304, -0.086)	<0.001	0.074 (0.030, 0.119)	0.001
**Combined pre-admission variables** [d]	-0.165 (-0.292, -0.038)	0.011	0.075 (0.022, 0.128)	0.006
**Reperfusion therapies** [e]	-0.147 (-0.269, -0.025)	0.02	0.073 (0.023, 0.124)	0.004
**Discharge medications** [f]	-0.200 (-0.311, -0.090)	<0.001	0.081 (0.036, 0.126)	<0.001
**Combined post-admission variables** [g]	-0.160 (-0.284, -0.036)	0.01	0.074 (0.023, 0.126)	0.004

a: Age, sex, smoking, diabetes, hypertension, dyslipidemia, history of stroke or TIA, atrial fibrillation, coronary artery disease, and congestive heart failure.
b: Age, sex, smoking, diabetes, hypertension, and dyslipidemia.
c: Antiplatelets, statins, and anticoagulants.
d: Age, sex, race, stroke risk factors, pre-stroke medications, time from symptom onset to admission, baseline mRS, and insurance.
e: Intravenous/intraarterial thrombolytics and stents.
f: Antiplatelets, statins, antihypertensives, anticoagulants, beta blockers, and ACEI’s/ARB’s.
g: Reperfusion therapies, discharge medications, and insurance.
NIHSS: National Institute of Health Stroke Scale, mRS: modified Rankin Scale
ADI: Area Deprivation Index

© 2022 Ghoneem A et al. *JAMA Network Open*.

eTable 7. Association Between Initial Stroke Severity Indices and Functional Outcome at 90 Days

Models with covariables	Initial stroke severity indices and mRS at 90 days (N=1015)			
	Infarct Volume		NIHSS	
	Standardized β (95% CI)	*p*	Standardized β (95% CI)	*p*
**NONE** (unadjusted)	0.412 (0.357, 0.467)	<0.001	0.607 (0.559, 0.655)	<0.001
**Age, sex, and race**	0.404 (0.352, 0.456)	<0.001	0.581 (0.534, 0.627)	<0.001
**Stroke risk factors** [a]	0.450 (0.395, 0.506)	<0.001	0.571 (0.525, 0.617)	<0.001
**Atherosclerotic risk factors** [b]	0.464 (0.408, 0.519)	<0.001	0.574 (0.528, 0.621)	<0.001
**Pre-stroke medications** [c]	0.412 (0.357, 0.466)	<0.001	0.606 (0.558, 0.654)	<0.001
**Time from symptom onset to admission**	0.396 (0.335, 0.457)	<0.001	0.610 (0.555, 0.666)	<0.001
**Baseline mRS**	0.466 (0.409, 0.524)	<0.001	0.587 (0.540, 0.634)	<0.001
**Insurance**	0.411 (0.357, 0.466)	<0.001	0.608 (0.559, 0.656)	<0.001
**Combined pre-admission variables** [d]	0.419 (0.357, 0.480)	<0.001	0.538 (0.485, 0.591)	<0.001
**Reperfusion therapies** [e]	0.463 (0.398, 0.528)	<0.001	0.643 (0.585, 0.700)	<0.001
**Discharge medications** [f]	0.379 (0.319, 0.439)	<0.001	0.546 (0.496, 0.596)	<0.001
**Combined post-admission variables** [g]	0.401 (0.334, 0.467)	<0.001	0.594 (0.536, 0.653)	<0.001

a: Age, sex, smoking, diabetes, hypertension, dyslipidemia, history of stroke or TIA, atrial fibrillation, coronary artery disease, and congestive heart failure.
b: Age, sex, smoking, diabetes, hypertension, and dyslipidemia.
c: Antiplatelets, statins, and anticoagulants.
d: Age, sex, race, stroke risk factors, pre-stroke medications, time from symptom onset to admission, baseline mRS, and insurance.
e: Intravenous/intraarterial thrombolytics and stents.
f: Antiplatelets, statins, antihypertensives, anticoagulants, beta blockers, and ACEI’s/ARB’s.
g: Reperfusion therapies, discharge medications, and insurance.
NIHSS: National Institute of Health Stroke Scale, mRS: modified Rankin Scale

© 2022 Ghoneem A et al. *JAMA Network Open*.

eTable 8. Association Between SES Measures and Functional Outcome at 90 Days Within the Subset of Patients Who Did Not Receive Reperfusion Therapies

Models with covariables	Median Income and mRS at 90 days (N=799)		ADI and mRS at 90 days (N=699)	
	Standardized β (95% CI)	*p*	Standardized β (95% CI)	*p*
**NONE** (unadjusted)	-0.096 (-0.162, -0.030)	0.005	0.038 (0.011, 0.065)	0.007
**Age, sex, and race**	-0.089 (-0.153, -0.025)	0.007	0.043 (0.017, 0.069)	0.001
**Stroke risk factors** [a]	-0.076 (-0.138, -0.015)	0.02	0.033 (0.008, 0.059)	0.01
**Atherosclerotic risk factors** [b]	-0.074 (-0.137, -0.011)	0.02	0.035 (0.009, 0.061)	0.008
**Pre-stroke medications** [c]	-0.094 (-0.160, -0.027)	0.005	0.037 (0.009, 0.064)	0.010
**Time from symptom onset to admission**	-0.093 (-0.167, -0.019)	0.01	0.036 (0.006, 0.066)	0.02
**Baseline mRS**	-0.087 (-0.151, 0.023)	0.008	0.038 (0.011, 0.064)	0.005
**Insurance**	-0.097 (-0.163, -0.031)	0.004	0.038 (0.010, 0.065)	0.007
**Discharge medications** [d]	-0.088 (-0.151, -0.025)	0.006	0.036 (0.011, 0.062)	0.006

a: Age, sex, smoking, diabetes, hypertension, dyslipidemia, history of stroke or TIA, atrial fibrillation, coronary artery disease, and congestive heart failure.
b: Age, sex, smoking, diabetes, hypertension, dyslipidemia.
c: Antiplatelets, statins, and anticoagulants.
d: Antiplatelets, statins, antihypertensives, anticoagulants, beta blockers and ACEI's/ARB's.
ADI: Area Deprivation Index, mRS: modified Rankin Scale

© 2022 Ghoneem A et al. *JAMA Network Open*.

eTable 9. Mediation Analysis Models With Multivariable Adjustments

Model covariables	Indirect paths	Standardized β (95% CI)	*p*	% of total effect of SES on functional outcome at 90 days
**NONE** (unadjusted) (n=993)	Income → Infarct vol → mRS	-0.0071 (-0.0143, -0.0014)	**<0.05**	7
	Income → NIHSS → mRS	-0.0396 (-0.0660, -0.0131)	**<0.05**	41
	Income → Infarct vol → NIHSS → mRS	-0.0197 (-0.0351, -0.0045)	**<0.05**	21
	**Combined indirect paths**	-0.0663 (-0.1017, -0.0314)	**<0.05**	**69**
**Age and sex (n=993)**	Income → Infarct vol → mRS	-0.0083 (-0.0157, -0.0018)	**<0.05**	8
	Income → NIHSS → mRS	-0.0364 (-0.0608, -0.0116)	**<0.05**	37
	Income → Infarct vol → NIHSS → mRS	-0.0184 (-0.0321, -0.0039)	**<0.05**	19
	**Combined indirect paths**	-0.0631 (-0.0955, -0.0288)	**<0.05**	**64**
**Age, sex, and race (n=993)**	Income → Infarct vol → mRS	-0.0086 (-0.0162, -0.0016)	**<0.05**	9
	Income → NIHSS → mRS	-0.0320 (-0.0557, -0.0088)	**<0.05**	35
	Income → Infarct vol → NIHSS → mRS	-0.0186 (-0.0324, -0.0033)	**<0.05**	20
	**Combined indirect paths**	-0.0591 (-0.0925, -0.0249)	**<0.05**	**64**
**Stroke risk factors [a] (n=991)**	Income → Infarct vol → mRS	0.0100 (-0.0194, -0.0018)	**<0.05**	13

© 2022 Ghoneem A et al. *JAMA Network Open*.

	Income → NIHSS → mRS	-0.0340 (-0.0569, -0.0121)	**<0.05**	41
	Income → Infarct vol → NIHSS → mRS	-0.0170 (-0.0313, -0.0031)	**<0.05**	20
	**Combined indirect paths**	-0.0610 (-0.0948, -0.0284)	**<0.05**	**74**
**Atherosclerotic risk factors [b] (n=991)**	Income → Infarct vol → mRS	0.0090 (-0.0181, -0.0011)	**<0.05**	11
	Income → NIHSS → mRS	-0.0332 (-0.0564, -0.0103)	**<0.05**	40
	Income → Infarct vol → NIHSS → mRS	-0.0167 (-0.0314, -0.0020)	**<0.05**	20
	**Combined indirect paths**	-0.0590 (-0.0923, -0.0242)	**<0.05**	**71**
**Model covariables**	**Indirect paths**	**β (95% CI)**	***p***	**% of total effect of Income on functional outcome at 90 days**
**Pre-stroke medications [c] (n=984)**	Income → Infarct vol → mRS	0.0070 (-0.0140, -0.0013)	**<0.05**	8
	Income → NIHSS → mRS	-0.0412 (-0.0666, -0.0155)	**<0.05**	44
	Income → Infarct vol → NIHSS → mRS	-0.0197 (-0.0346, -0.0045)	**<0.05**	21
	**Combined indirect paths**	-0.0679 (-0.1021, -0.0327)	**<0.05**	**73**
**Time from symptom onset to admission (n=792)**	Income → Infarct vol → mRS	0.0068 (-0.0146, -0.0008)	**<0.05**	8
	Income → NIHSS → mRS	-0.0420 (-0.0702, -0.0127)	**<0.05**	47

© 2022 Ghoneem A et al. *JAMA Network Open*.

	Income → Infarct vol → NIHSS → mRS	-0.0199 (-0.0364, -0.0022)	**<0.05**	22
	**Combined indirect paths**	-0.0687 (-0.1064, -0.0288)	**<0.05**	**77**
**Baseline mRS (n=991)**	Income → Infarct vol → mRS	0.0080 (-0.0160, -0.0014)	**<0.05**	9
	Income → NIHSS → mRS	-0.0353 (-0.598, -0.0116)	**<0.05**	40
	Income → Infarct vol → NIHSS → mRS	-0.0183 (-0.0332, -0.0035)	**<0.05**	21
	**Combined indirect paths**	-0.0617 (-0.0945, -0.0274)	**<0.05**	**70**
**Insurance (n=993)**	Income → Infarct vol → mRS	0.0071 (-0.0142, -0.0012)	**<0.05**	7
	Income → NIHSS → mRS	-0.0395 (-0.0656, -0.0143)	**<0.05**	41
	Income → Infarct vol → NIHSS → mRS	-0.0197 (-0.0349, -0.0039)	**<0.05**	21
	**Combined indirect paths**	-0.0662 (-0.1011, -0.0312)	**<0.05**	**69**
**Discharge medications** [d] **(n=990)**	Income → Infarct vol → mRS	0.0050 (-0.0113, -0.0005)	**<0.05**	5
	Income → NIHSS → mRS	-0.0383 (-0.0631, -0.0141)	**<0.05**	41
	Income → Infarct vol → NIHSS → mRS	-0.0173 (-0.0330, -0.0029)	**<0.05**	19
	**Combined indirect paths**	-0.0606 (-0.0927, -0.0285)	**<0.05**	**65**

a: Age, sex, smoking, diabetes, hypertension, dyslipidemia, history of stroke or TIA, atrial fibrillation, coronary artery disease, and congestive heart failure.
b: Age, sex, smoking, diabetes, hypertension, and dyslipidemia.

© 2022 Ghoneem A et al. *JAMA Network Open*.

c: Antiplatelets, statins, and anticoagulants.
d: Antiplatelets, statins, antihypertensives, anticoagulants, beta blockers, and ACEI's/ARB's.
SES: socioeconomic status, NIHSS: National Institutes of Health Stroke Scale, mRS: modified Rankin Scale

© 2022 Ghoneem A et al. *JAMA Network Open*.

eTable 10. Association Between SES Measures and Initial Stroke Severity Indices (Within the Subgroup of Patients With LAA and CE Strokes)

Models with covariables	Median income and initial stroke severity indices (N= 727)				ADI and initial stroke severity indices (N=621)			
	Infarct Volume		NIHSS		Infarct Volume		NIHSS	
	Standardized β (95% CI)	*p*	Standardized β (95% CI)	*p*	Standardized β (95% CI)	*p*	Standardized β (95% CI)	*p*
**NONE** (unadjusted)	-0.098 (-0.174, -0.022)	0.01	-0.157 (-0.232, -0.082)	<0.001	0.036 (0.004, 0.068)	0.03	0.078 (0.047, 0.109)	<0.001
**Age, sex, and race**	-0.097 (-0.173, -0.021)	0.01	-0.143 (-0.218, -0.068)	<0.001	0.036 (0.004, 0.068)	0.03	0.079 (0.049, 0.110)	<0.001
**Stroke risk factors** [a]	-0.089 (-0.157, -0.022)	0.009	-0.156 (-0.230, -0.083)	<0.001	0.038 (0.011, 0.065)	0.006	0.075 (0.045, 0.106)	<0.001
**Atherosclerotic risk factors** [b]	-0.081 (-0.148, -0.013)	0.02	-0.153 (-0.227, -0.078)	<0.001	0.036 (0.009, 0.064)	0.01	0.080 (0.050, 0.111)	<0.001
**Pre-stroke medications** [c]	-0.097 (-0.173, -0.021)	0.01	-0.155 (-0.230, -0.079)	<0.001	0.035 (0.003, 0.067)	0.03	0.078 (0.047, 0.110)	<0.001
**Time from symptom onset to admission**	-0.099 (-0.185, -0.014)	0.02	-0.143 (-0.232, -0.073)	<0.001	0.038 (0.002, 0.074)	0.04	0.075 (0.042, 0.108)	<0.001
**Insurance**	-0.098 (-0.174, -0.022)	0.01	-0.149 (-0.232, -0.082)	<0.001	0.036 (0.004, 0.068)	0.03	0.075 (0.042, 0.108)	<0.001

© 2022 Ghoneem A et al. *JAMA Network Open*.

**All covariables**	-0.078 (-0.151, -0.004)	0.04	-0.129 (-0.206, -0.051)	0.001	0.038 (0.008, 0.067)	0.01	0.074 (0.042, 0.106)	<0.001

a: Age, sex, smoking, diabetes, hypertension, dyslipidemia, history of stroke or TIA, atrial fibrillation, coronary artery disease, and congestive heart failure.
b: Age, sex, smoking, diabetes, hypertension, and dyslipidemia.
c: Antiplatelets, statins, and anticoagulants.
LAA: large artery atherosclerosis, CE: cardioaortic embolic, NIHSS: National Institute of Health Stroke Scale
ADI: Area Deprivation Index

© 2022 Ghoneem A et al. *JAMA Network Open*.

eTable 11. Association Between SES Measures and Functional Outcome at 90 Days (Within the Subgroup of Patients With LAA and CE Strokes)

Models with covariables	Median income and mRS at 90 days (N=667)		ADI and mRS at 90 days (N=570)	
	Standardized β (95% CI)	*p*	Standardized β (95% CI)	*p*
**NONE** (unadjusted)	-0.139 (-0.214, -0.063)	<0.001	0.049 (0.017, 0.081)	<0.001
**Age, sex, and race**	-0.136 (-0.206, -0.065)	<0.001	0.054 (0.024, 0.084)	<0.001
**Stroke risk factors** [a]	-0.115 (-0.184, -0.045)	0.001	0.042 (0.013, 0.071)	0.005
**Atherosclerotic risk factors** [b]	-0.114 (-0.185, -0.044)	0.001	0.045 (0.015, 0.075)	0.003
**Pre-stroke medications** [c]	-0.137 (-0.212, -0.061)	<0.001	0.048 (0.016, 0.080)	0.003
**Time from symptom onset to admission**	-0.136 (-0.220, -0.053)	0.001	0.048 (0.013, 0.084)	0.007
**Baseline mRS**	-0.122 (-0.195, -0.050)	0.001	0.045 (0.014, 0.076)	0.004
**Insurance**	-0.139 (-0.214, -0.064)	<0.001	0.049 (0.017, 0.081)	0.003
**Combined pre-admission variables** [d]	-0.104 (-0.178, -0.031)	0.005	0.037 (0.006, 0.068)	0.02
**Reperfusion therapies** [e]	-0.114 (-0.197, -0.032)	0.007	0.055 (0.021, 0.090)	0.002
**Discharge medications** [f]	-0.131 (-0.201, -0.062)	<0.001	0.049 (0.020, 0.078)	0.001
**Combined post-admission variables** [g]	-0.109 (-0.185, -0.033)	0.005	0.048 (0.016, 0.079)	0.003

© 2022 Ghoneem A et al. *JAMA Network Open*.

a: Age, sex, smoking, diabetes, hypertension, dyslipidemia, history of stroke or TIA, atrial fibrillation, coronary artery disease, and congestive heart failure.
b: Age, sex, smoking, diabetes, hypertension, and dyslipidemia.
c: Antiplatelets, statins, and anticoagulants.
d: Age, sex, race, stroke risk factors, pre-stroke medications, time from symptom onset to admission, baseline mRS, and insurance
e: Intravenous/intraarterial thrombolytics and stents.
f: Antiplatelets, statins, antihypertensives, anticoagulants, beta blockers, and ACEI’s/ARB’s.
g: Reperfusion therapies, discharge medications, and insurance.
NIHSS: National Institute of Health Stroke Scale, mRS: modified Rankin Scale, ADI: Area Deprivation Index

© 2022 Ghoneem A et al. *JAMA Network Open*.

eFigure 1. Distribution of Infarct Volume by Median Household Income

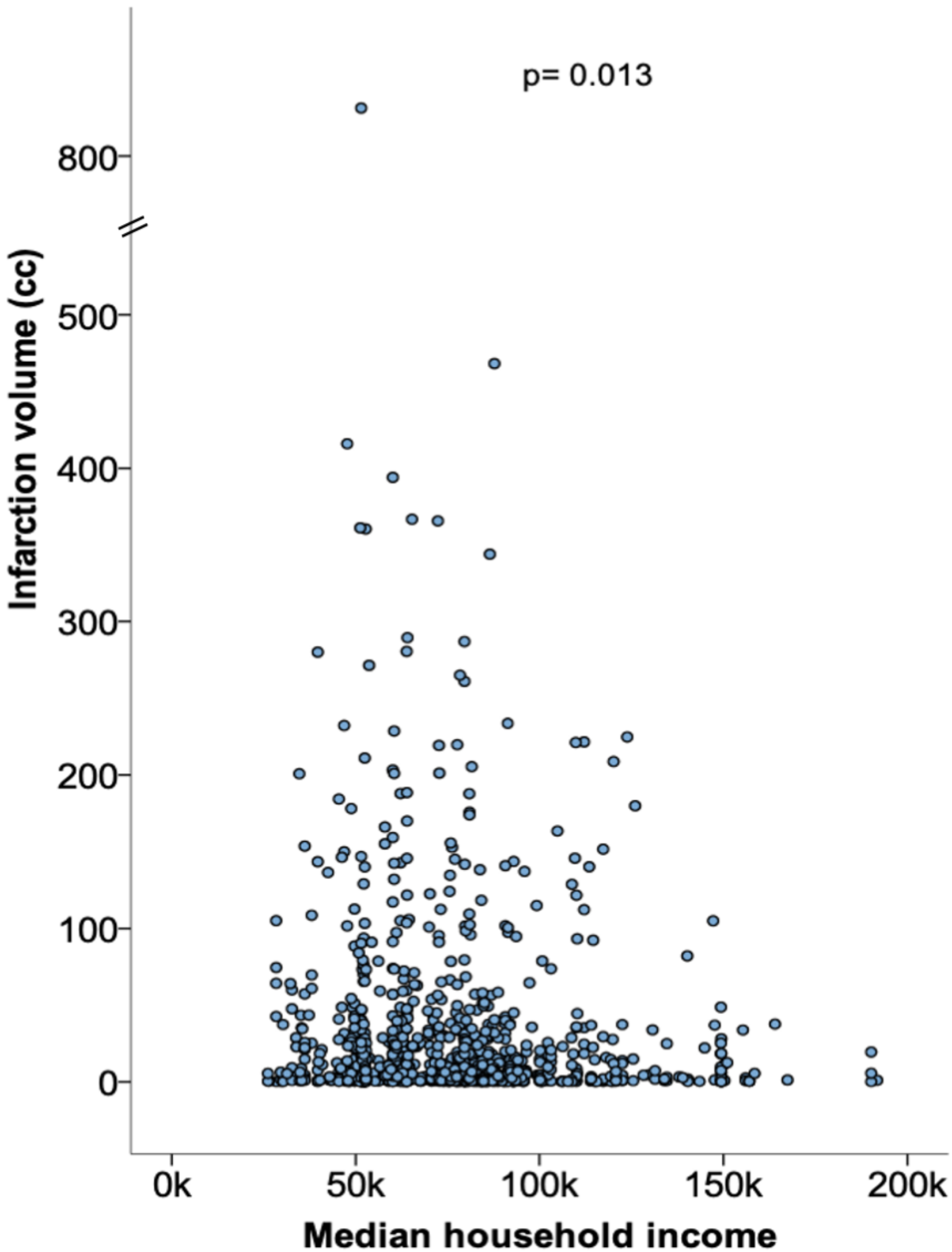

P value corresponds to the correlation coefficient -0.075

© 2022 Ghoneem A et al. *JAMA Network Open*.

eFigure 2. Association Between Different SES Measures (Income and ADI) and Study End Points

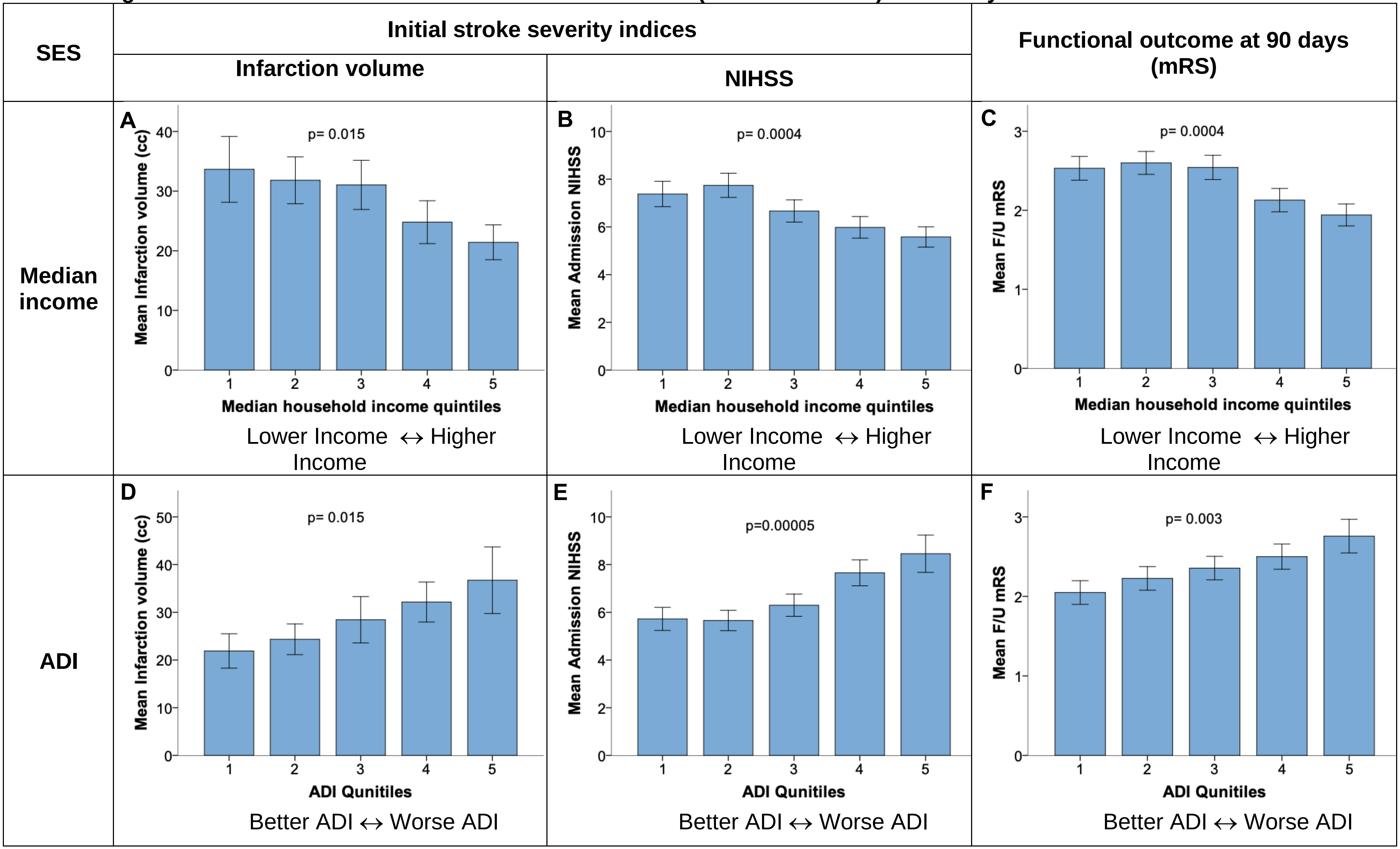

© 2022 Ghoneem A et al. *JAMA Network Open*.

A-C: Median income quintiles and: A) infarct size (infarct volume on MRI), B) clinical stroke severity on admission (NIHSS), C) functional outcome at 90 days (mRS).
D-F: ADI quintiles versus: D) infarct size (infarct volume on MRI), E) clinical stroke severity on admission (NIHSS), F) functional outcome at 90 days (mRS).
Error bars=1 standard error. NIHSS: National Institute of Health Stroke Scale, mRS: modified Rankin Scale. ADI: Area Deprivation Index.

© 2022 Ghoneem A et al. *JAMA Network Open*.

eFigure 3. Single-Mediator Mediation Analyses

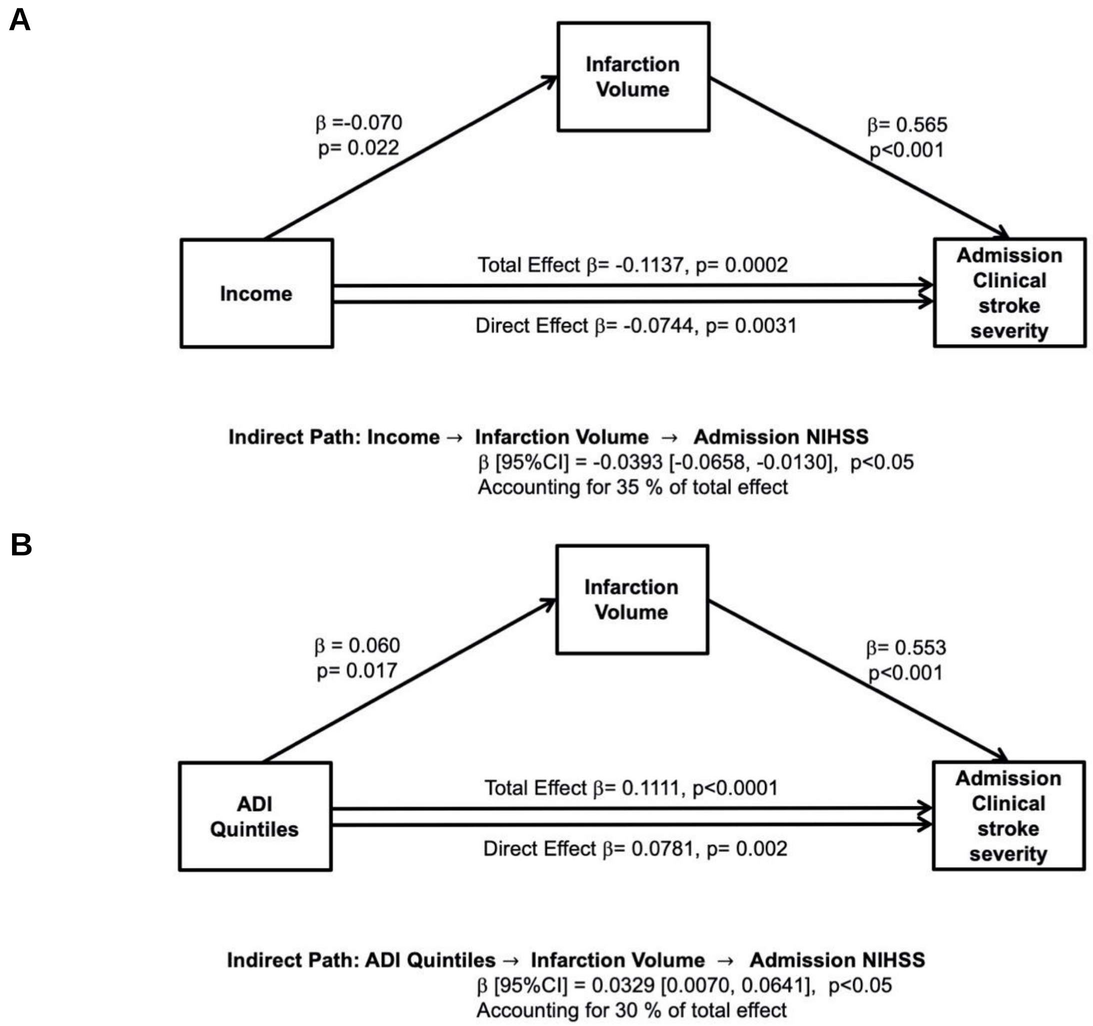

Single-mediator mediation analyses showing that infarct volume is a significant mediator of the relationship between SES (A: median income, B: ADI) and initial clinical stroke severity. The indirect pathway of lower income or higher ADI leading to larger strokes leading to more clinically severe strokes on admission was statistically significant in models adjusted for age and sex. NIHSS: National Institute of Health Stroke Scale, ADI: Area Deprivation Index.

© 2022 Ghoneem A et al. *JAMA Network Open*.

eFigure 4. A Serial 2-Mediator Mediation Analysis

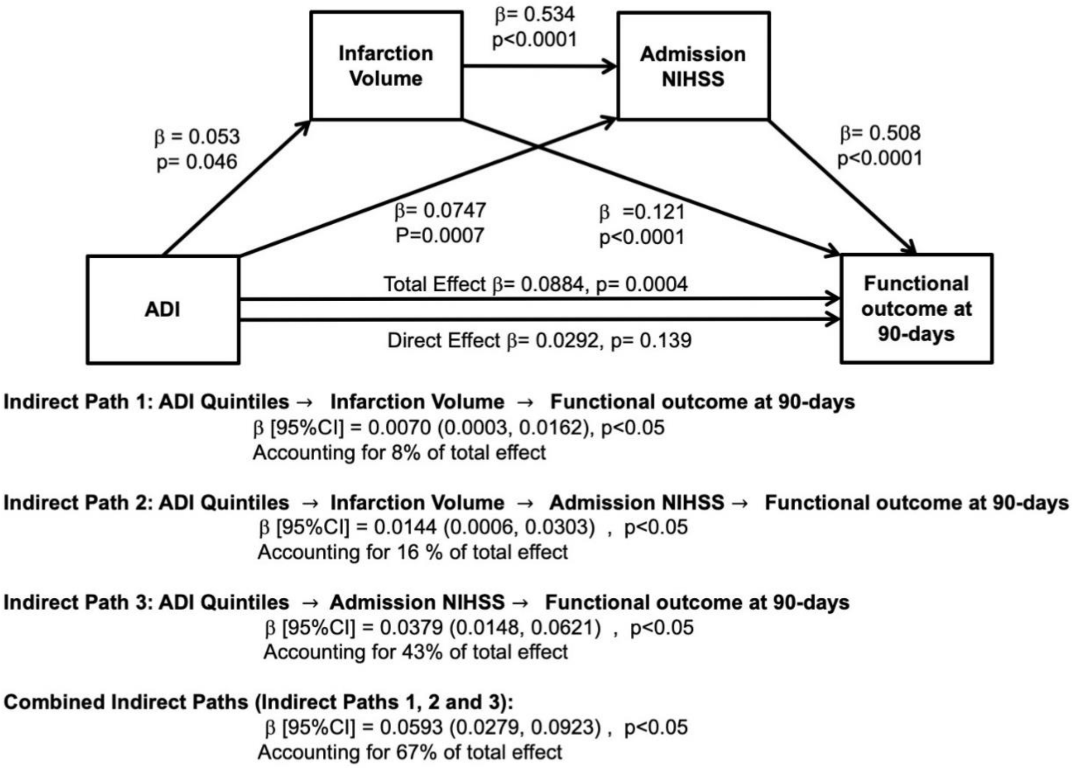

This model shows that initial stroke severity indices were significant mediators of the relation between ADI and functional outcome. Within this model, all indirect pathways involving both infarct volume or admission NIHSS (whether alone or in series) were statistically significant (adjusted for age and sex). Note that the direct effect was not statistically significant (i.e., excluding initial indices negated the association between SES and functional outcome).
ADI: Area Deprivation Index, NIHSS: National Institute of Health Stroke Sca

© 2022 Ghoneem A et al. *JAMA Network Open*.

eFigure 5. Association Between Different SES Measures (Income and ADI) vs Study End Points Within the Subgroup of Patients With LAA and CE Strokes

<table>
<tr><th rowspan="2">SES</th><th colspan="2">Initial stroke severity indices</th><th rowspan="2">Functional outcome at 90 days (mRS)</th></tr>
<tr><th>Infarction volume</th><th>NIHSS</th></tr>
<tr><td>Median income</td><td>A
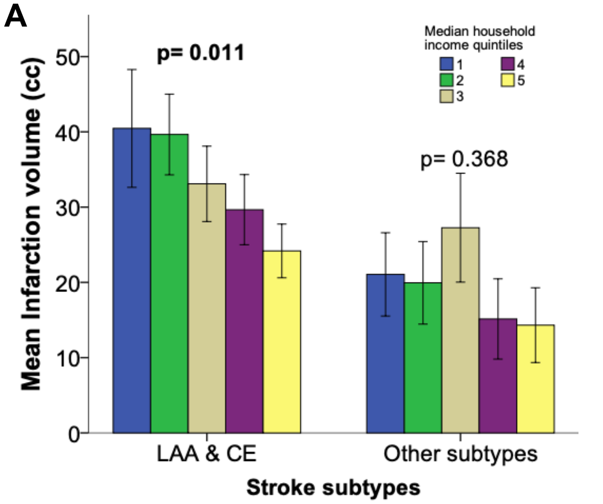

</td><td>B
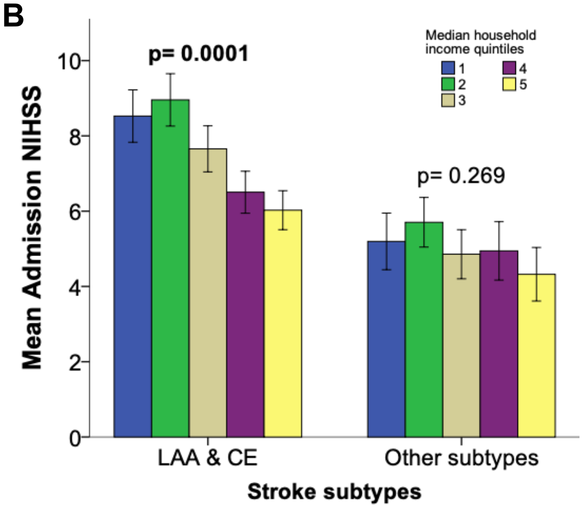

</td><td>C
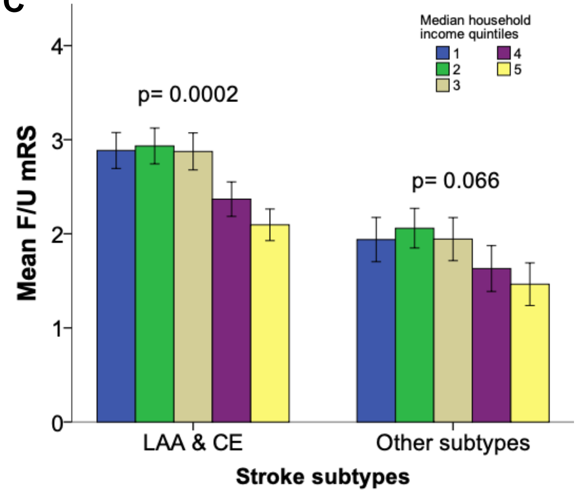

</td></tr>
</table>

D

E

F

© 2022 Ghoneem A et al. *JAMA Network Open*.

ADI

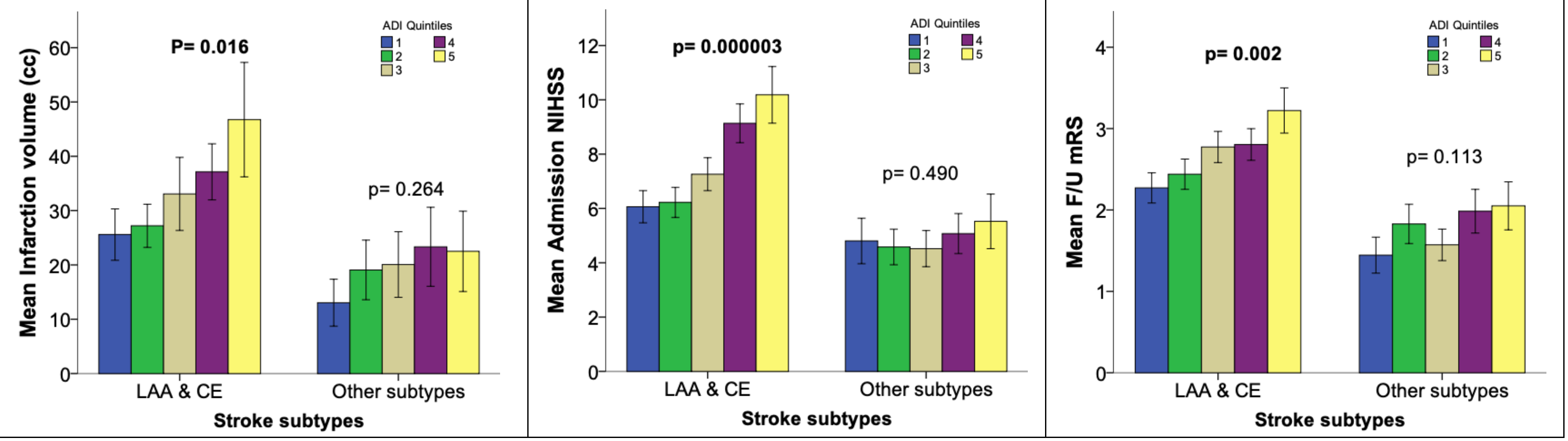

A-C: Median income quintiles and: A) infarct size (infarct volume on MRI), B) clinical stroke severity on admission (NIHSS), C) functional outcome at 90 days (mRS).
D-F: ADI quintiles versus: D) infarct size (infarct volume on MRI), E) clinical stroke severity on admission (NIHSS), F) functional outcome at 90 days (mRS).
Error bars=1 standard error. NIHSS: National Institute of Health Stroke Scale, mRS: modified Rankin Scale. ADI: Area Deprivation Index.

© 2022 Ghoneem A et al. *JAMA Network Open*.

eReferences

1. Singh GK. Area deprivation and widening inequalities in US mortality, 1969-1998. *Am J Public Health.* 2003;93(7):1137-1143.
2. Kind AJH, Buckingham WR. Making Neighborhood-Disadvantage Metrics Accessible - The Neighborhood Atlas. *The New England journal of medicine.* 2018;378(26):2456-2458.
3. Ay H, Arsava EM, Vangel M, et al. Interexaminer Difference in Infarct Volume Measurements on MRI. *Stroke.* 2008;39(4):1171-1176.
4. Ay H, Furie KL, Singhal A, Smith WS, Sorensen AG, Koroshetz WJ. An evidence-based causative classification system for acute ischemic stroke. *Annals of neurology.* 2005;58(5):688-697.
5. Ay H, Benner T, Arsava EM, et al. A computerized algorithm for etiologic classification of ischemic stroke: the Causative Classification of Stroke System. *Stroke.* 2007;38(11):2979-2984.
6. Arsava EM, Helenius J, Avery R, et al. Assessment of the Predictive Validity of Etiologic Stroke Classification. *JAMA neurology.* 2017;74(4):419-426.
7. Ay H, Arsava EM, Andsberg G, et al. Pathogenic ischemic stroke phenotypes in the NINDS-stroke genetics network. *Stroke.* 2014;45(12):3589-3596.
8. Arsava EM, Ballabio E, Benner T, et al. The Causative Classification of Stroke system: an international reliability and optimization study. *Neurology.* 2010;75(14):1277-1284.
9. Tawakol A, Ishai A, Takx RAP, et al. Relation between resting amygdalar activity and cardiovascular events: a longitudinal and cohort study. *The Lancet.* 2017;389(10071):834-845.
10. Introduction to Mediation, Modera- tion, and Conditional Process Analysis: A Regression-Based Approach [press release]. New York: Guilford Press2013.

© 2022 Ghoneem A et al. *JAMA Network Open*.
